# Machine Learning Study of Metabolic Networks *vs* ChEMBL Data of Antibacterial Compounds

**DOI:** 10.1021/acs.molpharmaceut.2c00029

**Published:** 2022-06-07

**Authors:** Karel Diéguez-Santana, Gerardo M. Casañola-Martin, Roldan Torres, Bakhtiyor Rasulev, James R. Green, Humbert González-Díaz

**Affiliations:** † Department of Organic and Inorganic Chemistry, University of Basque Country UPV/EHU, 48940 Leioa, Spain; ‡ Universidad Regional Amazónica IKIAM, Tena, Napo 150150, Ecuador; § Department of Coatings and Polymeric Materials, North Dakota State University, Fargo, North Dakota 58102, United States; ∥ Department of Systems and Computer Engineering, Carleton University, K1S5B6 Ottawa, Ontario, Canada; ⊥ BIOFISIKA, Basque Center for Biophysics CSIC-UPVEH, 48940 Leioa, Spain; # IKERBASQUE, Basque Foundation for Science, 48011 Bilbao, Biscay, Spain

**Keywords:** ChEMBL, information fusion, machine learning, antibacterial compounds, multidrug-resistant, complex networks, perturbation theory

## Abstract

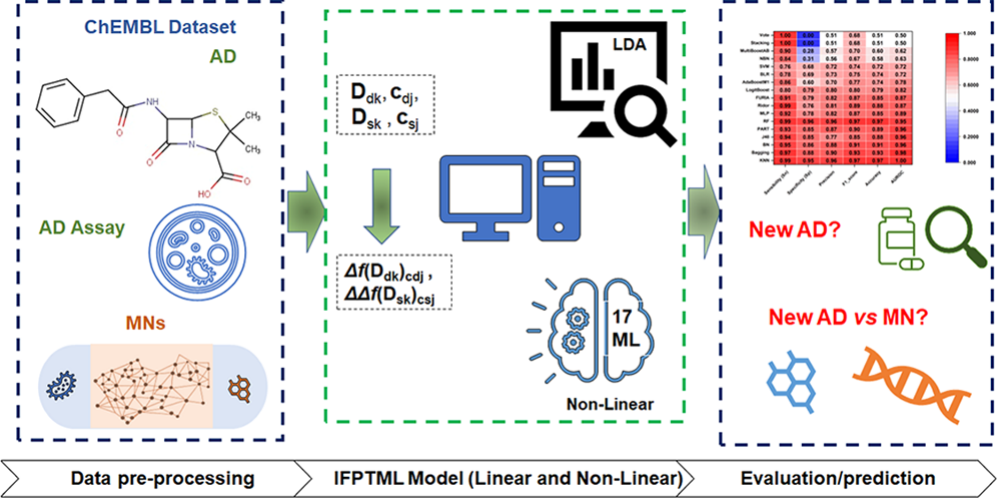

Antibacterial drugs (AD) change the metabolic status of bacteria, contributing to bacterial death. However, antibiotic resistance and the emergence of multidrug-resistant bacteria increase interest in understanding metabolic network (MN) mutations and the interaction of AD *vs* MN. In this study, we employed the IFPTML = Information Fusion (IF) + Perturbation Theory (PT) + Machine Learning (ML) algorithm on a huge dataset from the ChEMBL database, which contains >155,000 AD assays *vs* >40 MNs of multiple bacteria species. We built a linear discriminant analysis (LDA) and 17 ML models centered on the linear index and based on atoms to predict antibacterial compounds. The IFPTML-LDA model presented the following results for the training subset: specificity (Sp) = 76% out of 70,000 cases, sensitivity (Sn) = 70%, and Accuracy (Acc) = 73%. The same model also presented the following results for the validation subsets: Sp = 76%, Sn = 70%, and Acc = 73.1%. Among the IFPTML nonlinear models, the k nearest neighbors (KNN) showed the best results with Sn = 99.2%, Sp = 95.5%, Acc = 97.4%, and Area Under Receiver Operating Characteristic (AUROC) = 0.998 in training sets. In the validation series, the Random Forest had the best results: Sn = 93.96% and Sp = 87.02% (AUROC = 0.945). The IFPTML linear and nonlinear models regarding the ADs *vs* MNs have good statistical parameters, and they could contribute toward finding new metabolic mutations in antibiotic resistance and reducing time/costs in antibacterial drug research.

## 1 Introduction

Antibiotics have established themselves as the bedrock of modern medicine. However, the World Health Organization (WHO) in January 2017 produced a list of worldwide priorities for antibiotic-resistant microorganisms.^1^ The continued efficacy of antibiotics is jeopardized by the global spread of antibiotic resistance determinants, a process facilitated to a great extent by inappropriate use of antibiotics in clinical, community, and agricultural contexts.^2^ To design successful next-generation antibacterial medicines, we must first have a deeper understanding of how bacteria respond to antibiotics.^3^ Molecular screenings have identified compounds that limit bacterial growth *in vitro*. Despite the abundance of bioactive chemicals, only a few biological functions are targeted.^4^ Antibiotics that disrupt these energy-consuming pathways disrupt the metabolic balance.

Levy et al.^5^ proposed in 2004 that antibiotics have a finite duration of clinical value before being compensated for the inevitable emergence of resistance. Thus, new antibiotics are critical in fighting bacterial resistance.^6^ The majority of newly licensed antibiotics are chemically modified variants of established medication classes; several are found naturally.^7,8^ As a result, bacterial strains may rapidly evolve resistance mechanisms to these analogues if their existing resistance mechanisms do not already display partial cross-effectiveness.^9^

Furthermore, this bacterial resistance to conventional antibiotics has also been attributed to the excessive use of broad-spectrum antibiotics,^10^ which requires scientists to find fast, accessible, and cheap methods for discovering new drugs and target molecules against infectious microorganisms. In this regard, an understanding of pathogen metabolism is critical. Metabolic networks (MN) are made up of metabolic pathways, which are a series of biochemical reactions in which the result (output) of one reaction acts as a substrate (input) for another reaction.^11^ Novel applications of MN reconstructions of human pathogens have recently been described. These studies have focused on elucidating resistance metabolic dependencies and identifying potential drug targets and antibiotics.^12−14^ The influence of the changes in MNs on the capacity of various microorganisms to survive has been demonstrated by Barabási′s team and other authors.^15,16^

On the other hand, the importance of metabolic mutations in antibiotic resistance is frequently underestimated.^17^ Recently, Lopatkin et al.^18^ demonstrated that metabolic mutations arise in clinically relevant bacteria in response to antibiotic therapy. They used a variety of *in vitro* evolution procedures and comprehensive sequencing data analysis. The use of *Escherichia coli* as a model pathogen demonstrated that metabolic alterations can arise in response to antibiotic treatment.^18^ This research has provided a new perspective on the development of antibiotic resistance by shedding light on the complexities of metabolic alterations.^3^ Their findings may assist in explaining the prevalence of multidrug-resistant bacterial strains isolated in areas with little or no antibiotic exposure, as well as the documented increase in antibiotic resistance following extensive herbicide or other environmentally hazardous substance application.^18^ Antibacterial drugs (AD) change the metabolic status of bacteria, resulting in bacterial mortality, for example, through oxidative damage or stasis through translation inhibition, resulting in decreased cellular respiration.^3^ The bacterium’s metabolic state has an effect on antibiotic sensitivity; thus, altering the metabolic state can increase antibiotic efficacy.^3,17^ In this sense, the interaction of ADs and MNs can contribute toward finding new metabolic mutations in antibiotic resistance, mainly regarding (multi)drug-resistant bacteria.

On the other hand, prediction using computer models has been widely employed as a significant alternative to obtain experimental data and save resources and research time in drug discovery and development.^19,20^ These methods allow scientists to establish relationships between many datasets and structural molecular information that contributes to biological activity to solve complex problems.^21^ Additionally, machine learning (ML) enables us to process data in terms of molecular descriptors. Traditional methods for getting metadata from complex databases of preclinical assays are not good enough. One example of a traditional method is the ChEMBL database, which collects big datasets from a variety of heterogeneous and independent sources and aims to investigate complicated and dynamic interactions between data from preclinical trials.^22^

Numerous cheminformatics and other computational techniques have been developed to assist in the discovery of ADs against various bacteria. However, the techniques are limited to predicting the drugs’ biological activity in a certain strain under specified conditions.^23^ Multitask quantitative structure–biological effect relationship (mtk-QSBER) models have attempted to address these drawbacks.^24^ They allow the integration of multiple chemical and biological data types, enabling the simultaneous prediction of pharmacological activities, toxicities, and/or other safety concerns.^25^ Different approaches have been presented in the antibacterial field to estimate biological activities and the ADMET characteristics (absorption, distribution, metabolism, elimination, and toxicity) of diverse chemical compounds at the same time. For example, anti-Pseudomonas activity,^26^ antituberculosis effects,^27^ activity against bacteria present in noma^28^ or against Gram-negative bacteria,^29^ or to predict effective anti-staphylococcal agents.^30^ González-Díaz et al. developed IFPTML [Information Fusion (IF) + Perturbation Theory (PT) + Machine Learning (ML)],^31^ a technique for ML with multiple outputs and input-coded labels to address this type of challenge. The scoring function *f*(*s*_ij_)_calc_ is produced by the IFPTML model. IFPTML has been applied to complicated data analysis in molecular sciences,^31,32^ infectious disease,^33^ nanotechnology,^34,35^*etc*. Drugs, drug cocktails, proteins, genes, vaccines, MNs, and complex networks have all been implicated in these issues.^16,32,36−38^

**Figure 1:**
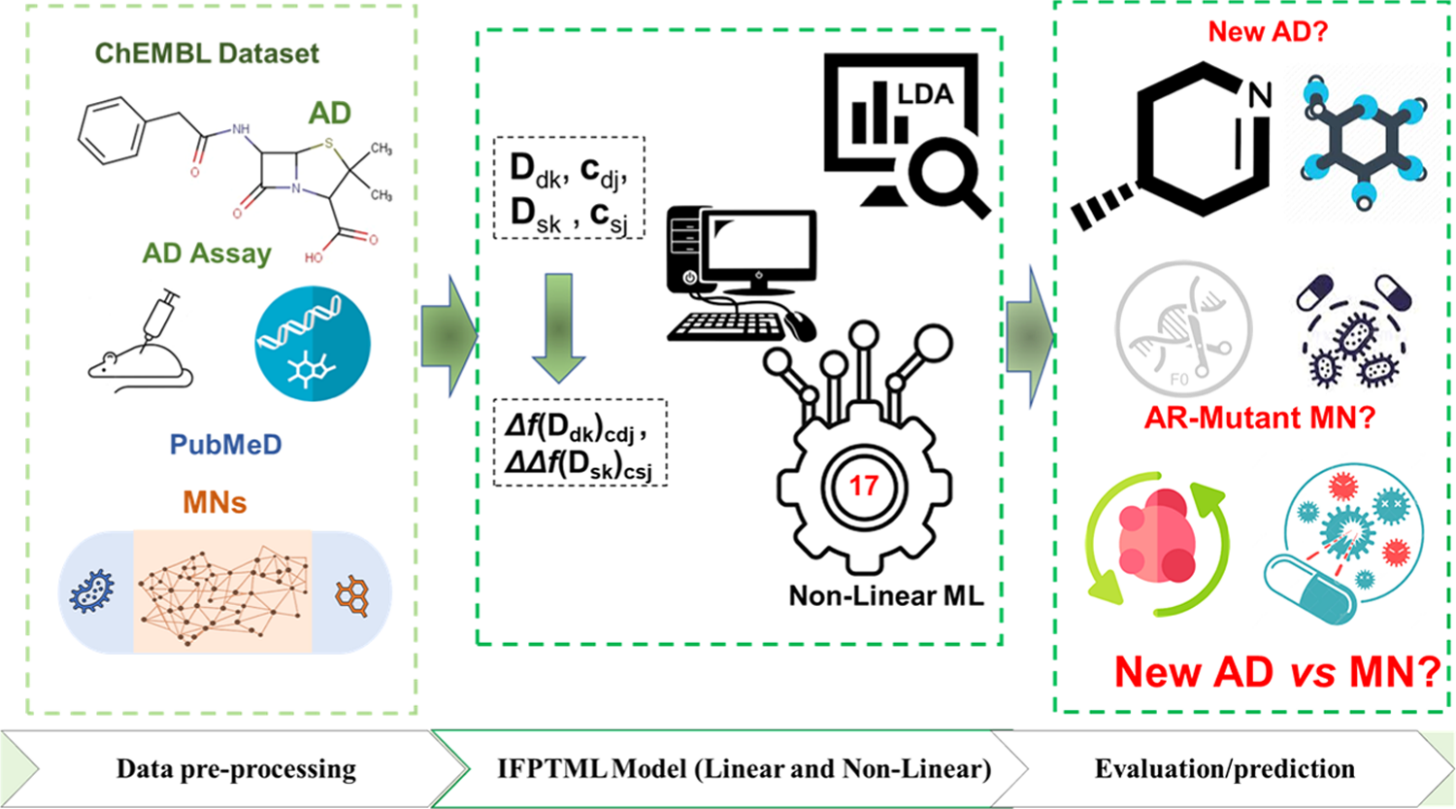
IFPTML model for AD *vs* MN development process.

The present study proposes a solution for this type of data by combining the basics of information fusion (IF), perturbation theory (PT), and machine learning (ML) approaches to create an IFPTML model.^35,39−42^ This paradigm is particularly well suited for databases with comparatively huge data characteristics and combinatorial information. This paper analyzed a large dataset (>155,000 preclinical assays) against different bacteria downloaded from the ChEMBL database. We merged this dataset with structural data for over 40 MNs from a variety of microorganisms previously reported by Barabási’s laboratory team.^15^ In all of these cases, those without biological values, measurements, or assay conditions were removed.^36^ We employed moving average (MA) operators to describe the assay perturbations and PT multiplier operators (PTOs) to achieve data combination and dimension reduction. Finally, we used linear discriminant analysis (LDA) and nonlinear ML algorithms to find the best IFPTML predictive model. Figure 1 illustrates the overall approach for developing the IFPTML model for ADs *vs* MNs.

## 2 Materials and Methods

### 2.1 ChEMBL Dataset of Antibacterial Compounds

We downloaded a large dataset of preclinical assays of ADs from the ChEMBL database. The dataset was created through a data fusion process between the ChEMBL dataset and Barabási’s group MNs released by Jeong et al.^15^ In this sense, we only searched in the ChEMBL database biological activity assays of ADs against organisms present in the MNs dataset. The steps carried out were as follows.

In the ChEMBL dataset, the organisms were searched for using targets and assays and saved in an Excel file. See details about this compound in Supporting Information **S00** (xlsx). Subsequently, we merged the datasets obtained with each keyword into a single file. Later, we performed the data curation, eliminating all duplicate cases and reporting no biological activity value. The data of the organisms *Methanococcus jannaschii* and *Treponema pallidum* were excluded since the two compounds reported in the ChEMBL have no biological activity measured. After data curation, we found that the ChEMBL AD activity dataset comprises values for >300 parameters (MIC, IC50, etc.) for >155,000 biological tests involving >50,000 compounds *vs* >25 bacteria species. Table S01 (Supporting Information **S01**) shows the statistics for multiple types of biological activity parameters in the ChEMBL dataset.

### 2.2 IFPTML Analysis Steps

**Figure 2:**
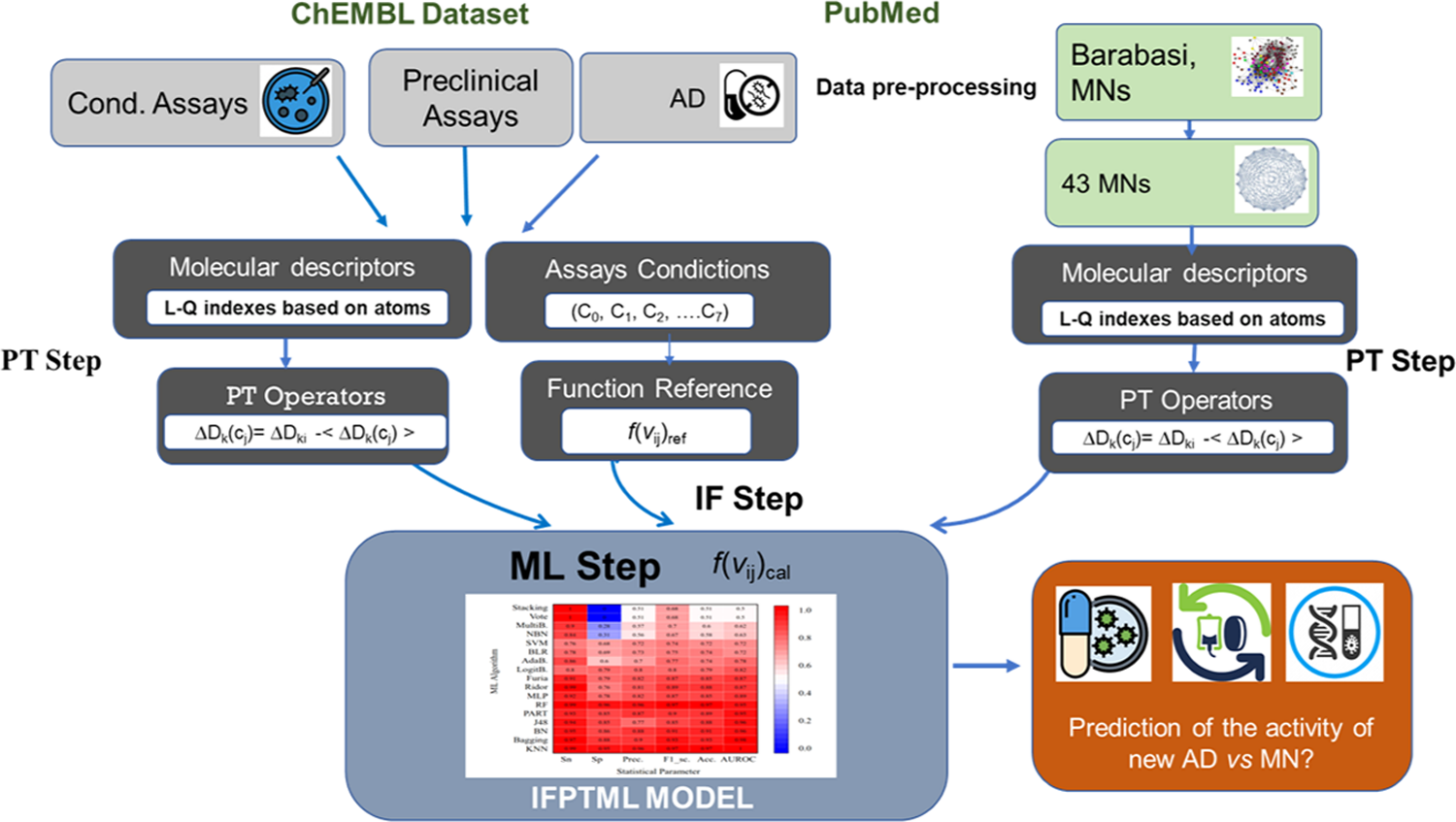
Detailed workflow diagram for IFPTML information processing.

The IFPTML analysis process is divided into three phases (IF + PT + ML). The IFPTML technique workflow for AD *vs* MN analysis is depicted in Figure 2, along with the general processes discussed in this research. The initial step in the IF phase is to obtain values *v*_i_ and *v*_j_ for the different biological properties c_d0_ and c_s0_ of the two subsystems (AD and MN). Following that, we preprocessed all observed values using a variety of units, scales, and degrees of uncertainty to create dimensionless functions that characterize the system as a whole, as well as the AD *vs* MN situations. Barabási’s group released the MN dataset as gzipped ASCII files.^15^ The numbers of nodes (metabolites), input–output links (metabolic reactions), node degrees, topological indices, names, and codes of >40 bacteria species analyzed here appear in Tables S02 and S03 (Supporting Information **S01**). In the IF approach, the chemical compounds’ structures of ADs (*f*_k_(D_i_) values) were fused with structural information included in the MN datasets of the various species. All instances were assigned to one of two series: training (subset = t) or validation (subset = v). Sampling should be random, representative, and stratified to the greatest extent practicable.^43^ To build triads, we randomly picked original data from the two datasets. Following that, cases were randomly assigned to set = t and set = v in proportions of 75 *vs* 25%.^43^ The total of 154,214 compounds were divided into 115,662 for the training set and 38,552 for the validation set.

The output *f*(*v*_ij_)_calc_ was determined as a linear combination of scores for several *c*_i_, which is a generic term that indicates a variety of multioutput assay circumstances, such as targets, assays, organisms, and MNs. Moreover, *c*_0_ is the biological activity *v*_ij_ minimal inhibitory concentration (MIC (μg·mL^–1^)) or minimal bactericide concentration (MBC (μg·mL^–1^)), *etc.;c*_1_ is the specific protein (ChEMBL database); *c*_2_ is the assay organism in the experiment; *c*_3_ is the MN microorganism species; *c*_4_ is the target type; and *c*_*6*_ comprises mappings to the ChEMBL targets. Table S04 in the Supporting Information **S01** contains more information. The parameters *f*_k_, Δ*f*_k_^i^(*c_q_*), and ΔΔ*f*_k_^i^(*c_q_*) are the independent input variables, while *f*(*v*_ij_)= 1 is the input dependent variable. The molecular descriptors, D_ik_, of linear indexes based on atoms include *f_q_* (*N*, *M*, *w*) *g* for each chemical *q*. Eq 1 shows the general definition of linear indexes based on atoms (eq 1).
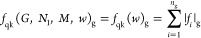
1where *N*_1_ is the selected matrix norm (Manhattan distance), *M* denotes the graph-theoretic electrical density matrix, and *w* denotes the physicochemical weight. In this scenario, the Ghose-Crippen Log P, the electronegativity, and the van der Waals volume were used. Finally, the following atom groups were estimated for the compounds H (A) bond acceptors, C atoms in the aliphatic chain (C), H link donors (D), C atoms in the aromatic part (P), and heteroatoms (X).^44^

Next, we defined and determined the values of all vectors corresponding to the structural descriptors D_dk_ and D_sk_ for the two subsystems. Additionally, we defined and calculated the vector elements c_dj_ and c_sj_ with all AD and MN bacteria labels/assay conditions. Following that, we transformed the estimated molecular descriptors D_dk_ and D_sk_ to Box–Jenkins MA operators. The PTOs estimated in this work include the chemical structure and/or physicochemical properties of the AD subsystem Δ*f*(D_dk_), as well as structural information about the bacteria’s MN ΔΔ*f*(D_sk_). They were written in the form of deviation terms for each subsystem *f*(D_dk_) and *f*(D_sk_) with relation to the average value for the respective subsystems of reference: ⟨*f*(D_dk_)_**c**dj_⟩ and ⟨*f*(D_sk_)_**c**sj_⟩. As a result, the initial terms *f*(D_dk_) and *f*(D_sk_) in these formulas denote the subsystem, while the averages denote the assay. The following equations were utilized (eqs 2 and 3).
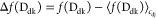
2
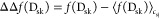
3In the Supporting Information S00, we detailed all fused datasets of drugs, and the PTO’s values of the IF technique (training, validation, and screening sets).

#### 2.2.1 IFPTML Linear Model

The IFPTML model was obtained from the merger of several cheminformatics methods. The IFPTML model output is the scoring function values *f*(*v*_ij_)_calc_ for the biological activity of the *i*th chemical assessed in the *j*th preclinical assay under the circumstances *c*_j_ = (*c*_0_, *c*_1_.... *C*_6_) against the sth bacterium species with MNs. The model begins with a reference value *f*(*v*_ij_)_ref_ and incorporates the influence of perturbations (PT operators) under the conditions of assay or the bacteria used, etc. The PT operators Δ*f*_k_ based on Box–Jenkins moving average (MA) operators have been utilized based on previously published studies to solve different problems.^38,45,46^ Linear discriminant analysis (LDA) was used to create the linear classification models. Equation 4 shows the general form of the IFPTML linear models.
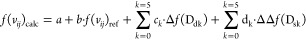
4The statistical parameter utilized to validate the model was the number of training examples (N), and the overall values of Model quality were determined using parameters such as sensitivity (Sn), specificity (Sp), Chi-square (χ^2^), and the p-level. LDA algorithms were run using the STATISTICA 6.0 program.^47^Figure 2 shows the IFPTML processing information in a detailed workflow.

#### 2.2.2 IFPTML Nonlinear Models

Next, we ran numerous nonlinear ML techniques built with the Waikato Environment for Knowledge Analysis (WEKA) software program, version 3.8.5.^48^ We employed a total of 17 ML methods to construct these different nonlinear IFPTML classification models using the current dataset. Classifiers such as Bayesian networks, decision trees, ensemble approaches, rule-based classifiers, neural networks, and functions were included in this category. Each strategy employs a learning algorithm to determine the model that most closely matches the relationship between the input dataset and the class. Based on Bayes’ theorem, the Bayesian Network K2/B (BN) and Nave Bayes network (NBN) classifiers were developed. The classification trees applied were Random Forest (RF)^49^ and the pruned or unpruned C4.5 decision tree classifier (J48).^50^ RF is an extension of Bagging, with the addition of randomized feature selection. It first selects a subset of features at random and then performs the traditional split selection technique inside the selected feature subset.^51^

Different ensemble methods were used. They include meta-algorithms that aim to combine weak learners’ skills such as bagging, boosting, voting, and stacking. In the first case, bagging methods are used to lower the variance of a base estimator (*e.g.*, decision tree) before constructing an ensemble from it. They are a quick and easy technique to improve a single model without changing the fundamental base algorithm.^51^ An implementation of CART (SimpleCart) was applied based on classifier trees in the Weka package.^52^ The second group is the boosting algorithms that are capable of transforming weak learners into strong ones. Intuitively, a weak learner does little better than a random guess, whereas a strong learner performs almost perfectly.^51^ In this work, we employed three exemplary algorithms from this family of algorithms: Adaboost, LogitBoost, and MultiBoosting.^53^ These models were built in conjunction with classifier trees based on entropy (DecisionStump). Voting is a straightforward ensemble procedure that generates two or more submodels. Each submodel generates predictions that are combined in some manner, such as by computing the mean or mode of the forecasts, allowing each submodel to vote on the appropriate conclusion.^54^ Finally, stacking is a universal technique that may be thought of as a straightforward expansion of voting ensembles, where an individual learner is combined. Individuals are considered first-level learners, while combiners are called second-level or meta-learners.^51^ In this work, the meta classifier ZeroR was used as the base model.

Artificial neural network (ANN) classification is a nonlinear classification technique inspired by biological neural networks. Feature vectors are used to describe objects (compounds). Each characteristic is associated with a weight and is transmitted to an input neuron. Input is routed to the output layer via hidden layers based on these weights.^55^ The output layer mixes these signals (*e.g.*, activity or class prediction). Weights are initially set at random. The weights are changed as the network is fed data so that the overall output approximates the observed endpoint values for the chemicals.^56^ In our work, the “hidden” layer was developed from 2 to 13 to predict the antibacterial compounds.

Other functions such as k nearest neighbors (KNN), binary logistic regression (BLR), and various support vector machines (SVMs) were implemented. KNN is a method for *lazy learning* that allocates novel compounds to the most prevalent class of known compounds in their near neighborhood,^57,58^ and numerous parameter combinations have been established. The number of the nearest neighbors (k) varied between 1 and 20. In addition, we employed the four distances (Chebyshev, Edit, Euclidean, and Manhattan) of the LinearNNSearch in a feature space. BLR is an algorithm that can be used for predicting a categorical variable (*e.g*., Yes/No or Pass/Fail) using a set of independent variables (*s*).^57,58^ Finally, SVM is a method that works well with noisy data.^59^ Identifying a stiff choice hyperplane that results in the highest potential margins across activity classes leads to models. Kernels can be used to translate nonlinear data to higher dimensions.

In the case of the rule-based classifiers, three methods were applied. PART is a decision list that constructs a partial C4.5 decision tree in each iteration and converts the best leaf to a rule;^60^ Ripple-Down Rule (Ridor) learner constructs a default rule and then the exceptions to the default with the lowest (weighted) error rate. The exceptions are a set of rules that forecast classes other than those picked by default,^61^ and Hühn and Hüllermeier proposed the Fuzzy Unordered Rules Induction Algorithm (FURIA), a revolutionary fuzzy rule-based categorization approach.^62^ The performance metrics used were Area Under Receiver Operating Characteristic (AUROC), Accuracy (Acc), Sn, Sp, Precision, and F1 score.

#### 2.2.3 Domain of Applicability (DoA)

Producing reliable forecasts necessitates an understanding of the model’s constraints and applicability. The DoA can be established using either the leverage approach or similarity metrics based on Euclidean distances between all training and test composites.^63,64^ We employed the leveraging technique. The residuals of the response variables were plotted against the leverages (the diagonal values of the hat matrix (*h*)) to visually define the DoA after computing the hat matrix for the structural domain (Williams plot).^65^ Chemicals that exceeded specified threshold values were identified as outliers in terms of reactivity and leverage. Three residuals were used as response thresholds. Leverage was used to set the critical hat value (h* = 3(*p* + 1)/*n*, where *p* denotes the number of model descriptors and *n* is the number of training compounds).^65^ Gramatica^66^ classified (*h* > h*) as a structurally significant chemical. In addition to testing series, the DoA was performed for an external series composed of 224,719 compounds (without antibacterial activity).

## 3 Results and Discussion

### 3.1 IFPTML Linear Model

The proposed IFPTML model is a synthesis of PTML modeling and information fusion (IF) techniques. The model begins with the predicted value of biological activity and then integrates the effects of various system disturbances. Two input variables are used in the model: the expected-value function *f*(_vij_)_ref_ and the Δ*f*, ΔΔ*f* PT operators. In Table 1, we show selected variables of the IFPTML-LDA model for different conditions used in the model. The criteria chosen are those that are expected to be more significant in terms of biological activity (AD *vs* MN).

**Table 1:** IFPTML Workflow Variables Model

phase	step	name	symbol	information	formula/description
IF	0	value	*v*_ij_	biological activity	value v_ij_ (MIC, MBC, *etc*.) of the parameter (labeled *c*_0_) determined for the *i*th compound under assay conditions cj = [*c*_0_, *c*_1_, *c*_2_ ... *c*_max_]
	1	objective function	*f*(*v*_ij_)_obs_	biological activity	*f*(*v*_ij_)_obs_ = 1 IF (*v*_ij_ > cutoff_j_ AND *d*(*c*_0_) = 1) OR (*v*_ij_ <cutoff_j_ AND *d*(*c*_0_) = −1) ELSE *f*(*v*_ij_)_obs_ = 0
					boolean variable obtained from the original biological activity value *v*_ij_
	2	reference function	*f*(*v*_ij_)_ref_	drugs chemical structure	*f*_14*q*_
					expected value of linear indices (C atoms in aliphatic chain/nonstochastic matrix order 2)
PT	3		Δ*f*_1_	drug structure *vs* protein accession	[*d*_14*q*_ – ⟨*d*_14*q*_(*c*_1*q*_)⟩]
					account for variability on linear indices (C atoms in aliphatic chain/nonstochastic matrix order 2) of the drug structure of metabolite *q* in the MN, under same conditions *c*_1_ (specific protein of the ChEMBL database)
			Δ*f*_2_	drug structure *vs* MN microorganism	[*d*_14*q*_ – ⟨*d*_14*q*_(c_4*q*_)⟩]
					account for variability on linear indices (C atoms in aliphatic chain/nonstochastic matrix order 2) of the drug structure of metabolite *q* in the MN, concerning MN Microorganism (*c*_4_)
			Δ*f*_3_	drug structure *vs* target mapping ChEMBL	[*d*_15*q*_ – ⟨*d*_15*q*_(*c*_7*q*_)⟩]
					account for variability on linear indices (C atoms in aliphatic chain/nonstochastic matrix order 3) of the drug structure of metabolite *q* in the MN, under conditions *c*_7_ (mappings to ChEMBL targets). It included different Target Mapping ChEMBL such as nonmolecular, protein unassigned, homologous protein, multiple proteins, multiple homologous proteins, homologous protein complex, molecular (nonprotein), protein complex.
			Δ*f*_4_	drug structure *vs* target type	[*d*_14*q*_ – ⟨*d*_14*q*_(*c*_5*q*_)⟩]
					account for variability on linear indices (C atoms in aliphatic chain/nonstochastic matrix order 2) of the drug structure of metabolite *q* in the MN, under conditions *c*_5_ (different target types). It included different types of ChEMBL targets as organism, single protein, unchecked, cell line, nucleic acid, protein complex, ADMET, protein family, no target, tissue, protein complex group, protein–protein interaction.
			Δ*f*_5_	drug structure *vs* protein accession	[*d*_15*q*_ – ⟨*d*_15*q*_(*c*_1*q*_)⟩]
					Account for variability on linear indices (C atoms in aliphatic chain/nonstochastic matrix order 3) of the drug structure of metabolite *q* in the MN, with respect to a specific protein in a ChEMBL database (*c*_1_).
			Δ*f*_6_	drug structure *vs* target type	[*d*_15*q*_ – ⟨*d*_15*q*_(*c*_5*q*_)⟩]
					account for variability on linear indices (C atoms in aliphatic chain/nonstochastic matrix order 3) of the drug structure of metabolite *q* in the MN, under the same conditions *c*_5_ (different target types).
	4		ΔΔ*f*_1_	metabolic network structure *vs* protein accession	[*d*_01*o*_ – ⟨*d*_01*o*_(*c*_1*o*_)⟩] – [*d*_01*s*_ – ⟨*d*_01*s*_(*c*_5*s*_)⟩]
					account for variability on linear indices (global indices/nonstochastic matrix order 1) of the query organism *o* and the organism of reference *s* in the MN, for the same specific protein in a ChEMBL database (*c*_1_).
			ΔΔ*f*_2_	metabolic network structure *vs* MN microorganism	[*d*_02*o*_ – ⟨*d*_02*o*_(*c*_3*o*_)⟩] – [*d*_02*s*_ – ⟨*d*_02*s*_(*c*_3*s*_)⟩]
					account for variability on linear indices (global indices/nonstochastic matrix order 2) of the query organism *o* and the organism of reference *s* in the MN, with respect to the same MN Microorganism (c_4_)
			ΔΔ*f*_3_	metabolic network *vs* MN microorganism	[*d*_03*o*_ – ⟨*d*_03*o*_ (*c*_3*o*_)⟩] – [*d*_03*s*_ – ⟨*d*_03*s*_(*c*_3*s*_)⟩]
					account for variability on linear indices (global indices/nonstochastic matrix order 3) of the query organism *o* and the organism of reference *s* in the MN, with respect to the same MN microorganism (*c*_3_)
			ΔΔ*f*_4_	metabolic network structure *vs* target type	[*d*_03*o*_ – ⟨*d*_03*o*_(*c*_4*o*_)⟩] – [*d*_03*s*_ – ⟨*d*_03*s*_ (*c*_4*s*_)⟩]
					account for variability on linear indices (global indices/nonstochastic matrix order 3) of the query organism *o* and the organism of reference *s* in the MN, with the same types of ChEMBL targets.
ML	5	output function	*f*(*v*_ij_)_calc_	score of biological activity	*f*(*v*_ij_)_calc_= *a* + *bf*(*v*_ij_)_ref_ + *c*_k_·Δ*f*(D_dk_) + *d*_k_·ΔΔ*f*(D_sk_)
					real valued output of the model
	6	predicted probability	*p(f*(*v*_ij_)_obs_ = 1)	score of biological activity	*p(f*(*v*_ij_)_obs_ = 1) = 1/(1 – (π_0_)/(π_1_))·exp(−*f*(*v*_ij_)_calc_))
					predicted probability of *f*(*v*_ij_)_obs_ = 1
	7	predicted class	*f*(*v*_ij_)_obs_	predicted class	*f*(*v*_ij_)_obs_*= 1 IF p(f*(*v*_ij_)_obs_ = 1)⟩ 0.5 ELSE *f*(*v*_ij_)_obs_ = 0
					predicted biological activity class

The probabilities used *a priori* to fit the model were set π_0_ (*f*(*v*_ij_= 0)) = π_1_(*f*(*v*_ij_= 1)) = 0.5. The molecular descriptors were transformed to Box–Jenkins moving averages. Two duplex linear indices atom-based level descriptors were used (with C atoms in an aliphatic chain and total (global)indices). In the first, nonstochastic matrix orders 2 and 3 were included in the model. In the second, the nonstochastic matrix order varied from 0 to 3 (more information is available in Table S5 in Supplementary Information **S01**). The output of the model *v*_ij_ is a score function for the biological activity of the *i*th AD under various combinations of the assay conditions *c*_sj_ and *c*_dj_. In this work, one chemical is classified as active based on its desirability d(*c*_0_) of the biological characteristic *v*_ij_(*c*_0_) and a preset cutoff value. The minimum inhibitory concentration (MIC) for biological activity *v*_ij_(*c*_0_) was set to be less than 4213 g·mL^–1^ or less than the average for unmeasured characteristics. When *v*_*ij*_> cutoff and the a priori desirability function *d*(*c*_0_) = 1, the AD was regarded to be active (*f*(*v*_ij_)_obs_ = 1). Additionally, if *v*_ij_<cutoff and *d*(c_0_) = −1, then *f*(*v*_ij_)_obs_ = 1; otherwise, (*f*(*v*_ij_))_obs_ = 0. When we aim to maximize the value of biological activity *s*_ij_(*c*_0_), such as inhibition (%), the desirability is *d*(c_0_) = 1. On the other hand, *d*(*c*_0_) = −1 when the value of biological activity *v*_ij_(*c*_0_) is desired to be minimized, for example, potency (nM), IC_50_ (nM), K_i_(nM), or EC_50_ (nM). Otherwise, when the necessity of maximizing or decreasing *v*_ij_(*c*_0_) is ambiguous, the value of desirability was taken to be *d*(*c*_0_) = 0. In any instance, the values of *d*(*c*_0_) for the same property can be changed (swapped) to suit a particular circumstance.^67^

Equation 5 contains a full explanation of the input variables analyzed, and the best model discovered has the following equation
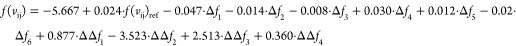
5
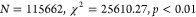
The model’s statistical parameters are as follows: *N* is the number of training examples, χ^2^ is the Chi-square statistics, and *p* is the *p*-level.

As shown in eq 5, the parameters Δ*f*_1_, Δ*f*_2_, Δ*f*_*3*_, Δ*f*_6_, and ΔΔ*f*_2_ all have a negative effect on the numerical score of the biological activity; these parameters correspond to the boundary conditions for the measure, target, and data curation. On the other hand, the variables *f*(*v*_ij_)_ref_, Δ*f*_4_, Δ*f*_5_, ΔΔ*f*_1_, ΔΔ*f*_3_, and ΔΔ*f*_4_ (protein, MN organism, and target type) all influence the activity positively. Additionally, we may obtain the parameters that contribute most to the activity using this equation. In the case of ΔΔ*f*_3_, the coefficient is 2.513, which is a very realistic value considering that the most significant variations in activity, even among identical compounds, are explained by the diverse techniques employed to assess the activity. The same holds true for the ΔΔ*f*_2_ parameter, which has a coefficient of 3.523 in the equation and contributes significantly in a negative way to activity.

Molecular descriptors enable the indirect correlation of desired attributes with a molecule’s structure.^68^ Analysis of the structural interpretation of the IFPTML-LDA model showed that total and local linear indices (atom and atom type) are the most influential descriptors in the chemical datasets, specifically nonstochastic matrix orders 2 and 3 in the presence of C atoms in the aliphatic chain. These can be aliphatic hydrocarbons with only single covalent bonds (alkanes), hydrocarbons that contain at least one C–C double bond (alkenes), and hydrocarbons that contain a C–C triple bond (alkynes). The T-Total (Global) indices are included in the nonstochastic matrix orders: 0, 1, 2, 3. The structural significance of these descriptors can be illustrated in several ways: as (a) chain length effect, (b) branching effect, (c) multiple bond effect, and (d) heteroatom modification effect.^69^ The influence of these structural characteristics on these molecular descriptors (which have mathematical linear map matrices) is referred to as graph-theoretic electronic structure models.^69^ Specifically, zero-order total (and local) linear indices can be classified according to their “dimensionality” as one-dimensional (1D) descriptors. These include “bulk” properties and physicochemical properties (hydrophobicity, molecular polar surface area, molar refractivity, molecular polarizability, and sum atomic charge).^70^ In general, these linear indices (total and local) include information about various structural changes in organic molecules, including chain-lengthening, branching, heteroatom content, and multiple bonds. However, most of the variables selected by the model only account for variability on linear indices (global indices/nonstochastic matrix).

The classification matrices for training and validation series are shown in Table 2. The results are summarized in terms of Sn = sensitivity (%), Sp = specificity (%), and Acc = accuracy (%). In both the training and validation series, the IFPTML-LDA model presented very high-performance parameters. The examples in the training and validation series were chosen using a stratified, random, and representative sampling technique. The obtained IFPTML model classified correctly ∼73% of the cases in the training and validation set. Both series have adequate values of sensitivity (Sn) and specificity (Sp), with ∼76%, and 70%, respectively. In general, the IFPTML model performed well at defining correct/incorrect connection patterns, as demonstrated by the performance of the current classification equation’s statistical parameters.

**Table 2:** IFPTML Linear Model Results for ChEMBL ADs *vs* MNs

series	set	stat. param^a^	%	*f*(*v*_ij_)_pred_ = 0	*f*(*v*_ij_)_pred_ = 1
training	*f*(*v*_ij_)_pred_ = 0	Sp	75.9	45,145	14,336
	*f*(*v*_ij_)_pred_ = 1	Sn	70.0	16,880	39,301
	Total	Acc	73.0		
validation	*f*(*v*_ij_)_pred_ = 0	Sp	76.0	15,066	4760
	*f*(*v*_ij_)_pred_ = 1	Sn	70.0	5621	13,105
	total	Acc	73.1		

a Sn = sensitivity (%), Sp = specificity (%), and Acc = accuracy (%). The positive (1) and negative control cases (0) were assigned as follows: if *a priori* desirability function *d* (c_0_) = −1, then *f*(*v*_ij_)_obs_ = 1 when *s*_ij_<cutoff. In addition, if *d*(c_0_) = 1, 0, then *f*(*v*_ij_)_obs_ = 1 when *v*_ij_> cutoff; otherwise, *f*(*v*_ij_)_obs_ = 0.

The IFPTML model was also generally adept at defining the correct/incorrect connection pattern, as demonstrated by the performance of the current classification equation’s statistical parameters.

### 3.2 IFPTML Nonlinear Models

**Figure 3:**
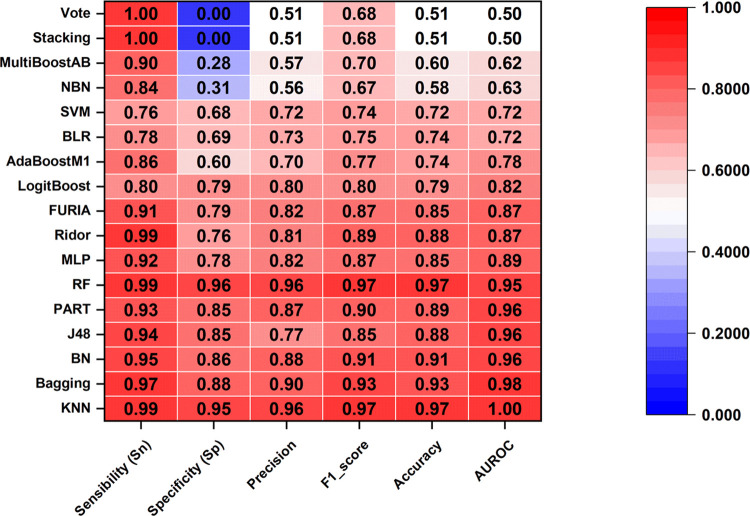
Detailed score for the training set considering 17 ML techniques applied.

**Figure 4:**
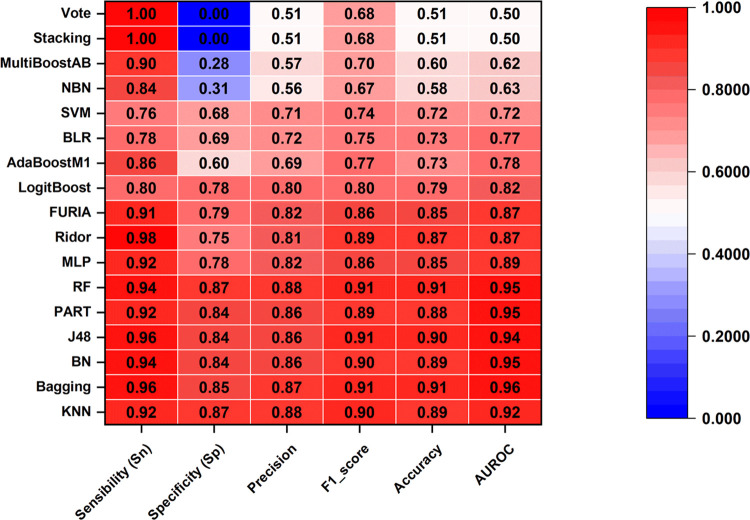
Detailed score for the test set considering 17 ML technique applied.

Additionally, we trained a different form of IFPTML model utilizing a distinct class of machine learning methods. We utilized a total of 17 machine learning classifiers. The performance of these models is summarized in Table 3, and the findings are displayed graphically in Figures 3 and 4.

**Table 3:** IFPTML Nonlinear AD *vs* MN Systems Models

				class	observed		
models^a^	subset^b^	stat.^c^	val. (%)	pred.	1	0	AUROC^d^
KNN	t	Sn	99.18	1	58,991	2549	0.998
		Sp	95.46	0	490	53,632	
	v	Sn	91.92	1	18,224	2446	0.924
		Sp	86.94	0	1602	16,280	
RF	t	Sn	98.63	1	58,669	2229	0.953
		Sp	96.03	0	812	53,952	
	v	Sn	93.96	1	18,628	2430	0.945
		Sp	87.02	0	1198	16,296	
Bagging	t	Sn	97.46	1	57,969	6722	0.982
		Sp	88.04	0	1512	49,459	
	v	Sn	95.86	1	19,005	2823	0.96
		Sp	84.92	0	821	15,903	
BN	t	Sn	95.48	1	56,791	7870	0.964
		Sp	85.99	0	2690	48,311	
	v	Sn	93.91	1	18,619	2970	0.947
		Sp	84.14	0	1207	15,756	
J48-DT	t	Sn	93.90	1	27,684	8160	0.958
		Sp	85.48	0	1797	48,021	
	v	Sn	96.00	1	2976	22,009	0.944
		Sp	84.11	0	15,750	16,543	
Part	t	Sn	93.06	1	55,352	8508	0.955
		Sp	84.86	0	4129	47,673	
	v	Sn	92.41	1	18,321	2972	0.946
		Sp	84.13	0	1505	15,754	
MLP	t	Sn	92.10	1	54,783	12,241	0.888
		Sp	78.21	0	4698	43,940	
	v	Sn	92.02	1	18,243	4138	0.885
		Sp	77.90	0	1583	14,588	
FURIA	t	Sn	91.31	1	54,315	11,705	0.871
		Sp	79.17	0	5166	44,476	
	v	Sn	91.45	1	18,131	3967	0.869
		Sp	78.82	0	1695	14759	
Ridor	t	Sn	98.67	1	58,687	13,452	0.874
		Sp	76.06	0	794	42,729	
	v	Sn	98.31	1	19,490	4615	0.868
		Sp	75.36	0	336	14,111	
LogitBoost	t	Sn	79.87	1	47,506	12,025	0.819
		Sp	78.60	0	11,975	44,156	
	v	Sn	79.84	1	15,830	4078	0.817
		Sp	78.22	0	3996	14,648	
AdaBoost	t	Sn	86.14	1	51,234	22,219	0.783
		Sp	60.45	0	8247	33,962	
	v	Sn	85.98	1	17,047	7527	0.782
		Sp	59.80	0	2779	11,199	
BLR	t	Sn	77.91	1	46,343	17,405	0.722
		Sp	69.02	0	13,138	38,776	
	v	Sn	77.99	1	15,463	5867	0.769
		Sp	68.67	0	4363	12,859	
SVM	t	Sn	76.09	1	45,257	17,959	0.721
		Sp	68.03	0	14,224	38,222	
	v	Sn	76.02	1	15,072	6050	0.719
		Sp	67.69	0	4754	12,676	
MultiBoostAB	t	Sn	89.56	1	53,274	40,450	0.623
		Sp	28.00	0	6207	15,731	
	v	Sn	89.57	1	17,759	13,482	0.622
		Sp	28.00	0	2067	5244	
NBN	t	Sn	84.04	1	49,988	38,683	0.628
		Sp	31.15	0	9493	17,498	
	v	Sn	84.02	1	16,657	12,900	0.629
		Sp	31.11	0	3169	5826	
Stacking (ZeroR)	t	Sn	100.00	1	59,481	56,181	0.5
		Sp	0.00	0	0	0	
	v	Sn	100.00	1	19,826	18,726	0.5
		Sp	0.00	0	0	0	
Vote	t	Sn	100.00	1	59,481	56,181	0.5
		Sp	0.00	0	0	0	
	v	Sn	100.00	1	19,826	18,726	0.5
		Sp	0.00	0	0	0	

a ML-Classification Models. kNN = k nearest neighbors, RF= Random Forest, Bagging, BN= Bayes network, J48-DT=J48 decision tree, Part, MLP = Multi-Layer Perceptron. FURIA = Fuzzy Unordered Rules Induction Algorithm, Ridor = RIpple-DOwn Rule, LogitBoost, AdaBoost, BLR = Binary Logistic Regression, SVM = Support Vector Machines, MultiBoostAB, NBN = Naïve Bayes, Stacking (ZeroR), and Vote.

b Subset. t =: Training set, v = Validation set.

c Stat. Statistical performance. Sn = Sensibility, Sp = Specificity.

d AUROC: Area under ROC value.

As expected, almost 10 of the 17 ML models displayed better Sn and Sp values than the IFPTML-LDA model. They are KNN, RF, Bagging, BN, J48-DT, Part, MLP, FURIA, Ridor, and LogitBoost. However, AdaBoost, BLR, SVM, MultiBoostAB, NBN, Stacking (ZeroR), and Vote showed a lower value of Sp than the IFPTML-LDA model. In the case of Stacking (ZeroR), Vote (Sn = 0%), and AUROC = 0.5, it indicates that classification is no better than random guessing. Thus, these techniques are not suitable for AD *vs* MN data processing. In terms of accuracy, the first 10 algorithms mentioned also presented good performance, with a global Acc = 80–97.4%, suggesting that this dataset herein is predominated by nonlinear classification.

Otherwise, in the validation set, the same algorithms KNN, RF, Bagging, BN, J48-DT, Part, MLP, FURIA, Ridor, and LogitBoost are higher than the IFPTML-LDA model. In addition, these techniques display satisfactory goodness of fit and goodness of prediction. They regularly outperform on both training and validation sets (see Table 3). The Sn rates for active and inactive classes are very high, indicating a significant discriminant capacity for future virtual screening applications.

In the training/validation set, the KNN, Bagging, BN, J48, PART, and RF show AUROC>0.95. The ROC curve is formed by graphing the true-positive rate versus the false-positive rate at various thresholds. Values close to 1 indicate that classification is almost perfect across all thresholds; thus, these six techniques are considered good classifiers for a dataset. They are the most accurate models as determined by a consensus examination of their general Acc and AUROC parameters. Nevertheless, the gain in performance from LDA to ML models was modest, and finding a model suitable for virtual screening assays is challenging.

#### 3.2.1 Domain of Applicability (DoA)

**Figure 5:**
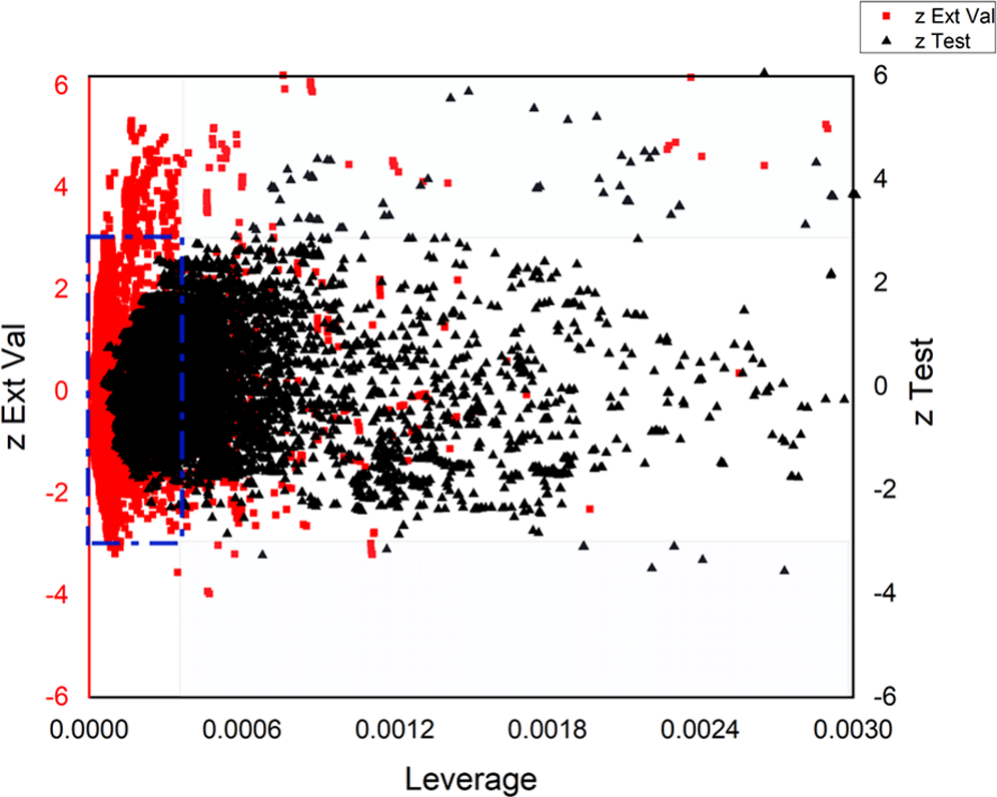
William’s plot (residuals *vs* leverages) for AD *vs* MN in the test and external validation sets.

The DoA of the IFPTML-LDA model is illustrated in Figure 5, as a double ordinate plot of residuals test sets (first ordinate) and plot of residuals external validation (second ordinate) *vs* leverages (abscissa) (William Plot). Within the domain, the examples fall within a rectangular area defined by a band of two residuals and a leverage threshold of *h* = 0.00033.^71^ As can be observed, the majority of validation examples fall inside this range. There are, however, a significant number of examples with leverage greater than the threshold but with standard residuals under the limits. In these instances, where the leverage value is greater than h*, the prediction should be regarded as untrustworthy. Values greater than the warning leverage (h*) indicate that the composite’s expected reaction can be extrapolated from the model, and hence the predicted value should be used with extreme caution. As a result, there are no instances in either the training or prediction series where the residual values are greater than the range defined for residuals and residual LOO. As a result, there are no outliers reported and our model is capable of accurately predicting new chemicals in this DoA.

### 3.3 Comparison with Other Heterogeneous Series of Compounds Approaches

The linear and nonlinear IFPTML of the ADs *vs* MNs were compared with other reports based on a heterogeneous series of compounds previously described in the literature in regard to discovering antibacterial compounds. Table 4 shows a comparison between the present model and some of these models (heterogeneous series of compounds, drug family >10). An analysis of Table 4 reveals that the current work has the greatest dataset (very complex and notably larger dataset in the number of compounds). Only six previous models contain more than 10,000 molecules. Compared to previous models with a parameter count of 6–8, the model provided in this study has a considerable number of parameters (12). However, **models 3**, **5**, and **6** show a greater number of variables: 62^72^ and 21,^73^ respectively.

**Table 4:** Chemoinformatic Approaches for the Development of Novel Antibacterial Compounds (Heterogeneous Series of Compounds, Drug Family >10)

model^a^	n^b^	act.^b^	var.^b^	tech.^c^	acc (%)	val^d^	multispecies^e^	MO^f^	net^g^	ref^h^
1	667	363	7	LDA	92.9	i	no	no	no	(78)
2	2030	1006	8	LDA	90.4	i	no	no	no	(79)
3	4346	520	62	kNN	95	ii	no	no	no	(72)
4	11,576	4208	4	ANN	97	i	*ST*	yes	no	(23)
5	7517	2066	21	kNN	99.3	i	*MRSA*	yes	no	(73)
6	7517	2066	21	SVM	92.9	i	*MRSA*	yes	no	(73)
7	37,834	13,203	5	LDA	95	i	No	yes	no	(77)
8	2230	1051	3	LDA	86.3	i	No		no	(80)
9	30,181	12,474	6	LDA	90	i	*FN*/*PI*	yes	no	(28)
10	54,682	19,912	6	ANN	90	i	*PS*	yes	no	(26)
11	3500	628	4	ISE	94.6	i	MBS	yes	no	(74)
12	74,567	8724	6	SOM	75.5	i	*EC*	yes	no	(75)
13	83,605	10,030	6	LDA	88.6	i	MBS	yes	yes	(76)
14	115,662	42,209	12	LDA	74.3	i	MBS	yes	yes	this work
15	115,662	42,209	12	kNN	97.4	i	MBS	yes	yes	
16	115,662	42,209	12	RF	97.4	i	MBS	yes	yes	

a Number of the model.

b *n* = The total number of cases included in the training and/or validation series. Act = Active drugs, and Vars. = Variables in the model.

c Technique: LDA = Linear discriminant analysis, KNN = K nearest neighbor, ANN = artificial neural network, SVM = support vector machine, ISE = iterative stochastic elimination, SOM = self-organizing map (Kohonen), RF = Random Forest.,.

d Val: validation methods. (i) external predicting series, test set, (ii) 100-times-averaged resubstitution technique.

e Multispecies: MBS = multiple bacterial strain, MRSA = methicillin-resistant *Staphylococcus aureus*, FN = Fusobacterium necrophorum, PI = Prevotella intermedia, EC = *E. coli*, PS = Pseudomonas spp, SS = Streptococcus spp.

f MO = multioutput: Models with multiple outputs can predict more than one sort of biological activity (MIC, IC50, MBC, etc.).

g =MNs: Models that can account for changes in the MNs of various microorganisms.

h Reference.

The LDA predominates among the techniques used to realize the models (6 of the 13). Two models include KNN (**model 3** and **5**)^72,73^ and ANN (**model 4** and **10**).^23,26^ Even though SVM is analyzed in one model (**model 6**),^73^ and the iterative stochastic elimination (ISE),^74^ and self-organizing map (SOM) (Kohonen)^75^ in the **models 11** and **12**, respectively. In the case of accuracy, it is worth noting that all models compared had precision values of more than 75%. However, the accuracy values of the RF and KNN techniques in this study (97.4%) are higher than those of other studies carried out with similar datasets, such as Nocedo et al.^76^ (88.6%). The external predicting series was the most frequently used validation technique in 12 of 13 models, including this one. This demonstrates that we used a time-tested validation technique. As illustrated in Table 4 (**models 1–3**, **7**, and **8**), the models are not able to predict multiple species; rather, they only predict one type of microorganism. Recently, multispecies models have been developed; some of them predict biological activity exclusively for members of the same genus or subgroup of bacteria (**models 4** to **13**), except for models **11**(74) and **13**,^76^**14–16**, which include multiple bacterial strains. Among them, the IFPTML models of our study (**models 14–16**) encompass the highest number of compounds and best accuracy (only **model 7**(77) is superior, but this work included only 7,517 compounds). In addition, our models include the prediction of antibacterial activity against various bacteria, including their MNs, which was only analyzed in **model 13**.^76^

## 4 Conclusions

The use of broad-spectrum antibiotics has been linked to bacterial resistance to conventional antibiotics. Understanding pathogen metabolism is critical for developing new medications and targets for antibacterial treatment. The impact of alterations in metabolic networks on the ability of various bacteria to survive has been demonstrated. In this research work, we developed an IFPTML-LDA model for predicting the antibacterial activity, which took into account the structure of MNs. The antibacterial activity and appropriateness of >155,000 biological experiments of >50,000 chemicals *vs* >25 different types of bacteria species were predicted using IFPTML-LDA models. Compared to other ML linear and nonlinear models (*e.g*., *SOM* models) presented in this work and in the literature, the model demonstrated strong predictive power (Sn, Sp, and Acc = 74%). Among the 17 ML algorithms employed in the development of nonlinear IFPTML classification models, the KNN, Bagging, BN, J48, PART, and RF models showed the highest AUROC, Accuracy, F1 score, Sn, and Sp values (>85% in training/validation sets). We can conclude that the IFPTML model stated could be a simple, valuable, and flexible tool, reducing time and costs in antibacterial drug investigation.

## Supporting Information Available

Statistics for multiple types of biological activity parameters in ChEMBL dataset (Table S01); details of the metabolic networks of >40 organisms (Table S02); average values of *f*_k_ for the metabolic networks of >40 organisms (Table S03); conditions included in ChEMBL dataset of antibacterial drugs vs MRN analysis (Table S04); and linear index based on atoms descriptors included in the model (Table S05) (PDF)
Search of organisms in the ChEMBL dataset using targets and assays (XLSX)
