# Supplementary material for: Machine Learning Study of Metabolic Networks vs ChEMBL Data of Antibacterial Compounds
Source: Mol Pharm. 2022 Jun 7;19(7):2151–63. doi: 10.1021/acs.molpharmaceut.2c00029 (PMC9986951; doi:10.1021/acs.molpharmaceut.2c00029)
Supplement: Supplementary file 2 — mp2c00029_si_002.pdf [file mp2c00029_si_002.pdf]

Table S01. Statistics for multiple types of biological activity parameters in ChEMBL dataset

Activity (units)	nj	avgj	sdj	α_j
MIC(ug.mL-1)	165796	4213.2	441434.8	-1
IC50(nM)	44656	212786866402.6	14602856035661.2	-1
IZ(mm)	20379	14.1	11.1	1
MIC(nM)	17880	1146514.9	12467958.5	-1
Inhibition(%)	15844	41.7	46.7	1
Activity(%)	11423	58.0	238.2	1
Ki(nM)	10351	1393209.4	20412213.9	1
MIC90(ug.mL-1)	8369	34.3	102.3	-1
Potency(nM)	8355	17495.1	10357.0	-1
MIC50(ug.mL-1)	5295	21.9	79.4	-1
Ratio()	4506	22361.1	1489387.6	1
CC50(nM)	3584	26964.4	19368.4	-1
IC50(ug.mL-1)	2944	1644.0	30542.8	-1
GI(%)	2630	52.3	37.4	1
MBC(ug.mL-1)	2570	199.4	1109.6	-1
Kd(nM)	2406	729636.1	15462331.9	-1
ED50(mg.kg-1)	2091	67.4	1123.4	-1
MIC90(nM)	2057	48219.5	230240.8	-1
PD50(mg kg-1)	1904	21.1	37.0	-1
Km(nM)	1715	3242907.5	44784769.3	1
Activity()	1671	4325.9	127964.5	1
Selectivity index()	1473	1039.5	26169.5	1
EC50(nM)	1463	176756.7	2689263.2	-1
Fold change()	1340	74896.5	2729750.7	1
no-measure	1260	3.1	13.4	1
MIC99(ug.mL-1)	1223	137.7	166.3	-1
IC90(nM)	1221	69341.6	54804.2	-1
Ratio IC50()	1213	860.5	6940.3	1
Relative Growth()	1195	-0.1	0.3	1
Kcat(s-1)	1050	2962.8	44588.1	1
AC50(nM)	1008	42232.5	71420.8	-1
FC()	881	5.6	24.1	1
Activity(ug.mL-1)	736	92.7	367.6	1
MIC99(uM)	664	512.5	10490.7	-1
Log10 cfu()	516	4.8	2.7	1
Kcat/Km(uM-1 s-1)	515	73.1	374.7	1
Ratio Ki()	503	292.0	2728.9	1
MIC(ug)	439	33.4	41.1	-1
Activity(uM)	431	159.4	913.5	-1
Activity(mM)	430	22.5	13.8	1
MBC(uM)	414	172.2	218.6	-1
Inhibition zone(mM)	403	19.7	10.9	1
Time(hr)	370	4412.7	14737.3	1
AbsAC40_uM(uM)	365	5.7	5.4	-1
Log 1/C()	332	5.1	1.4	1
fT>MIC(%)	322	49.0	32.7	1

deltaTm(degrees C)	320	4.1	7.1	1
log10CFU/ml()	315	8.4	79.3	1
MBC(ug ml-1)	312	68.3	139.3	-1
IC90(ug.mL-1)	307	57.9	76.2	-1
MIC99(nM)	297	46736.4	74659.0	-1
Log 1/MMIC()	296	2.3	0.8	1
Tm(degrees C)	285	17.8	25.7	1
ED50(nM)	280	245429.3	742587.1	-1
IC12(ug.mL-1)	279	480.7	1253.5	-1
Kinact(nM)	276	4373.9	30166.8	1
MIC50(nM)	275	12967600.8	137820917.9	-1
Average IC90(uM)	273	37.6	40.9	-1
MPC(ug.mL-1)	261	9.5	18.2	-1
Kcat(min-1)	257	26.7	196.3	1
MIC(ug cm-2)	252	14.9	13.4	-1
MIC(10'-2 umol/ml)	235	11.0	12.6	-1
MBC(10'-2 umol/ml)	235	16.6	19.0	-1
MPC8(ug.mL-1)	214	35.3	51.1	-1
log10cfu()	210	3.1	1.9	1
log(1/MIC)()	204	-0.4	1.8	1
MIC80(ug.mL-1)	202	77.7	91.8	-1
MIC>90(ug.mL-1)	198	22.0	30.5	-1
Efficacy(%)	191	48.2	35.7	1
MIC=>80(ug ml-1)	178	3.2	8.1	-1
MFC(ug.mL-1)	175	366.3	725.0	-1
Log2 MIC()	170	6.6	4.2	1
Growth_index()	167	84.3	283.1	1
TI()	163	116.1	249.0	1
PI()	162	3547.1	22053.2	1
Activity(mg)	162	62.2	152.0	-1
MNEC(ug.mL-1)	160	9.4	29.0	-1
Selectivity ratio()	156	836.4	9542.4	1
MBC(nM)	147	71180.1	116329.8	-1
Inhibition(mM)	144	9.1	5.7	1
deltalog10CFU/ml()	144	4.9	1.7	1
IZ(mm2)	140	60.8	73.3	-1
INH(uM)	140	38.1	43.1	-1
Log 1/Ki app()	137	6.6	1.4	1
Activity(nmol/min)	136	425.1	1166.7	-1
Post antibiotic effect(hr)	135	3.8	3.4	1
FICI()	132	1.4	6.0	1
Kcat(/s)	131	254.4	1335.7	-1
Max salt tolerance()	126	0.7	0.2	1
MIC95(ug.mL-1)	125	68.9	134.5	-1
MBC(uM/ml)	122	0.1	0.0	-1
Activity(ug)	121	85.2	195.7	-1
MTD(g/acre)	120	25.3	25.0	-1
Activity(nM)	117	38473.1	118937.7	1

MIC(ug mg-1)	117	9.2	13.0	-1
Kcat/Km()	117	4142737.3	19623331.0	1
Time(min)	110	11.1	14.5	1
CFU(ml-1)	109	8839.6	18940.2	-1
MLC(ug.mL-1)	107	2723.6	4463.5	-1
Concentration(ug.mL-1)	104	91.2	273.1	-1
Kcat/Km(mM-1 s-1)	104	583.6	1581.9	1
MBC90(ug.mL-1)	103	26.6	50.8	-1
Log10 CFU/ml()	100	3.0	2.6	1
ED50(mg kg-1 x 2)	100	16.8	12.7	-1
MIC(uM ml-1)	99	0.2	0.2	-1
log(1/C)()	98	3.7	1.4	1
MBC(uM ml-1)	98	0.4	0.3	-1
T/C()	93	5.7	12.2	1
Survival(%)	91	66.9	36.7	1
MIC=>90(ug.mL-1)	91	21.9	24.5	-1
-Log C(mM L-1)	88	7.2	0.8	1
Kcat/Km(M-1.s-1)	87	121755.3	466460.0	-1
MIC80(nM)	86	200392.4	1822460.7	-1
Drug uptake(ug.mL-1)	84	4.4	5.8	-1
MIC=>90(uM)	84	26.8	42.4	-1
Drug uptake(nmol/min)	84	1.7	1.1	-1
No. of Colony forming Units()	82	163632.6	577727.4	1
Drug metabolism(%)	81	16.9	37.1	1
fAUC/MIC()	80	74.8	144.6	1
T1/2(hr)	77	4.8	11.4	1
Kii(nM)	77	4431.7	9196.7	-1
MIC=>90(ug ml-1)	77	6.0	0.8	-1
MBC(mg/L)	74	35.3	34.5	-1
Drug metabolism(pmol/min)	74	3.2	2.1	-1
Drug uptake(%)	73	31.7	11.6	1
MIC95(uM)	73	154.6	217.3	-1
IZ(nM)	73	15.7	8.0	-1
Log Ki()	72	7.1	1.7	-1
MIC()	71	78.3	196.1	-1
Imax(%)	71	55.8	29.7	1
Ratio AUC/MIC()	71	36.1	80.4	1
MBC50(ug.mL-1)	70	12.9	16.1	-1
MIC100(ug.mL-1)	70	22.8	67.7	-1
MIC(ppm)	70	237.3	356.4	-1
RBA(%)	70	56.9	58.8	1
Inhibition(uM)	70	97.6	134.5	-1
Revertants/plate()	70	185.7	295.5	1
IC95(nM)	69	333814.5	195910.8	-1
MBC(umol/ml)	69	0.4	0.2	-1
Max activation(%)	68	307.1	170.0	1
EC90(uM)	65	53.4	42.6	-1

MIC>90(uM)	64	49.2	27.4	-1
Radius of inhibition zone(mM)	64	3.6	4.9	1
Ratio(M-1.s-1)	64	198901.4	1029090.8	1
Ratio EC50()	61	30778.4	192685.9	-1
EC50(ug.mL-1)	61	28.5	113.1	-1
MBC99.9(ug ml-1)	60	230.4	76.1	-1
Smax(%)	60	107.5	14.2	1
No. of revertants per plate()	60	43.7	54.0	-1
CFU()	59	655554.6	2999917.5	-1
CC25(ug.mL-1)	59	55.6	89.6	-1
Kcat/Km(10'6/M/s)	59	3.7	2.2	-1
Kis(uM)	59	1735.3	8505.9	-1
Stabilty(nmol/min)	58	65.2	187.8	1
MIC(10'-2umol)	58	26.3	4.9	-1
MBC(10'-2umol)	58	51.4	9.6	-1
Emax()	58	0.7	0.2	-1
DIZ(mm mg-1)	57	13.8	2.9	-1
CFU(uL-1)	57	758925.6	1478855.6	-1
KA(uM)	57	29.8	33.6	-1
Activity(ug ml-1)	56	3.9	7.3	-1
Phytotoxicity(%)	56	28.8	38.6	1
ED20(gAi/ha)	55	636.1	729.0	-1
Log D50()	54	1.4	0.7	1
Kis(nM)	54	774.9	1854.7	-1
MED(g/acre)	54	2.2	5.9	-1
Inhibition(radii mm-1)	52	9.8	10.4	1
Time(s)	52	110.6	85.6	1
MLC(ug ml-1)	51	150.7	72.6	-1
AUC(ng.hr.mL-1)	51	7807876220.3	22207669549.9	-1
pIC90()	51	4.5	0.5	1
Avg IC90(uM)	50	11.1	21.0	-1
Inhibitory diameter(mM)	49	23.2	4.9	1
ED50(mg kg-1 day-1)	49	19.9	26.5	-1
Inhibition zone(cm)	48	3.1	2.9	1
Activity(radii mm-1)	48	7.0	8.6	1
Inhibition concentration(ug.mL-1)	48	52.9	46.9	1
Activity(/nl)	48	1471.3	1805.4	1
pLADY()	48	5.9	0.8	-1
MIC(uL/ml)	47	329.9	335.4	-1
ID50(nM)	47	65596.0	68085.6	-1
Kcat/Km(10'5/M/s)	47	4.5	2.6	-1
MBC(mg ml-1)	46	3.2	5.7	1
Log SOSIP()	46	3.6	0.9	1
IZ(%)	46	29.0	41.7	1
Vmax(pmol/min)	46	6.5	9.0	1
ED50(ug.mL-1)	45	20.9	48.7	-1

log10CFU/g()	45	4.7	1.9	1
Cmax(ug.mL-1)	45	14.6	30.2	-1
MBC99.9(ug.mL-1)	45	106.1	78.4	-1
k cat(s-1)	45	43.8	257.6	1
Growth Index()	45	16.3	18.9	1
Survived(%)	44	44.1	48.3	1
CD50(mg kg-1)	43	102.5	330.1	-1
Log M50()	43	1.2	0.9	-1
IC80(uM)	42	26.5	23.8	-1
Kcat/Km(10'6/s/M)	42	2.3	1.2	-1
Inhibition(ug.mL-1)	42	5.1	15.1	-1
pK50()	41	5.5	0.5	1
XI50(molar excess)	41	31.8	45.2	-1
Relative potency()	40	3.5	2.2	1
CC50(ug.mL-1)	40	4.1	17.7	-1
C50(nM)	40	6100.8	14593.0	-1
Binding affinity()	40	63.7	46.3	1
Kcat(/min)	40	9.3	10.0	1
Mutagenicity()	40	166.2	196.8	-1
GI50(nM)	39	38659.7	161273.0	-1
MIC(umol ml-1)	39	6.4	13.2	-1
MBC(umol ml-1)	39	11.5	21.8	-1
MIC95(uM/ml)	39	8.1	7.0	-1
TBMIC(ug.mL-1)	39	2.6	2.6	-1
ED50(uM)	38	27.1	36.5	-1
k_off(s-1)	38	34.7	72.4	1
Vmax(nmol/min/mg)	38	1496.9	1387.2	1
D50(10'-5M)	38	14.8	17.5	-1
RatioAUC/MIC()	37	293.2	457.2	1
Growth zone(mM)	37	4.8	9.1	1
Survived()	37	5.9	3.6	1
pMIC()	37	1.9	1.1	1
MIC95(umol/L)	36	82.2	153.7	-1
Kcat/Km(/mM/s)	36	24.3	60.3	-1
LC50(nM)	35	21765.5	29822.5	-1
Uptake at C50()	35	4.7	1.9	1
MAC(nM)	35	257750.0	188143.1	-1
MPC4(ug.mL-1)	35	7.3	8.7	-1
MBC99.9(uM)	34	0.0	0.0	-1
Antibacterial activity()	34	0.6	0.6	1
Relative rate of hydrolysis()	34	202.9	286.4	1
LD90(nM)	34	2151.2	3801.6	-1
Kill at C50(%)	34	71.4	22.5	1
Kinact(/mM/min)	34	1523.9	2355.1	1
Cp(ug.mL-1)	34	9.9	18.5	-1
MIC=>80(uM)	32	60.0	0.0	-1
ID50(ug.mL-1)	31	358.1	656.3	-1
DIZ(%)	31	28.0	12.5	1

ID50(umol/L)	31	89.7	45.8	-1
Cleavage(ug.mL-1)	31	5.0	10.0	1
Emax(%)	31	77.1	40.0	1
Kcat/Km(uM/hr)	31	2.8	4.3	-1
MBIC(uM)	30	60.4	38.5	-1
Zone of stimulation(mM)	30	19.5	8.4	1
Log 1/MIC(M)	30	0.9	1.1	1
pMIC(M L-1)	30	1.8	1.9	1
Activity(fmol)	30	337.3	518.0	-1
Kiuc(uM)	30	70.8	76.1	-1
MIC>90(ug ml-1)	30	4.7	11.6	-1
Kcat(app)(/min)	30	7.9	7.5	-1
log(M50)()	30	0.4	0.8	-1
GI(ug ml-1)	29	1.7	2.3	-1
MED(mg kg-1)	29	36.2	29.5	-1
EC>90(uM)	29	1.8	5.4	-1
Diameter of inhibition zone(mM)	29	25.7	11.9	1
MIC70(ug.mL-1)	28	53.1	81.1	-1
MIC(ug cm-3)	28	7.2	2.5	-1
GI50(ug.mL-1)	28	2.5	3.6	-1
Kdiss(uM)	28	3328.9	4789.1	1
Kic(uM)	28	21.0	13.6	-1
Ratio(ug.mL-1)	28	19.1	57.9	-1
Activity(log10CFU/ml)	28	2.5	1.0	1
Tmax(hr)	27	2.1	1.7	1
ZImax/KZI(mm microL/micromol)	27	3.1	7.2	1
%max(%)	27	93.1	24.0	1
EC90(ug.mL-1)	27	1.3	3.2	-1
EC99(ug.mL-1)	27	2.9	3.7	-1
IC99(nM)	27	52554.1	42473.9	-1
FIC50()	27	0.5	0.6	-1
Ks(nM)	26	47710.2	93265.2	-1
logMIC()	26	1.9	1.2	1
ED50(mg mouse-1)	26	4.2	4.0	-1
CFU/ml()	26	75004.8	254750.2	-1
Km(uM-1 s-1)	26	180.2	229.0	-1
MED(g)	26	82.2	27.3	-1
GR50(ppm)	25	272.2	315.7	-1
EC1.5(nM)	25	12112.0	27152.0	-1
GI99(ug ml-1)	25	15.3	19.3	-1
Activity(degree)	25	47.2	29.2	-1
I90(ppm)	25	189.6	213.6	-1
MIC100(ug ml-1)	24	21.4	26.4	-1
Relative potency(fold)	24	1.1	2.1	1
Activity(mm3)	24	1231.1	601.6	-1
Log TA98()	24	3.4	0.9	-1

Activity(Miller_unit)	24	336.2	143.3	-1
Log k()	23	4.2	2.3	1
Kcat(%)	23	25.6	29.7	1
Kcat/Km(uM-1 min-1)	23	1.3	3.2	1
Activity(micromol/min)	23	6.3	13.1	-1
Inhibition_index()	23	385.3	273.3	1
Solubility(%)	23	160.8	314.3	1
MIC99(ug ml-1)	23	40.9	52.6	-1
deltalog10CFU()	23	0.7	1.7	1
LD50(uM)	22	30.8	35.7	-1
MCC(nM)	22	12727272.7	10501270.4	-1
EII50()	22	15.9	17.9	-1
Ka(M-1)	22	819959090.9	1297636526.3	1
Kii(uM)	22	39.0	117.3	-1
Ratio(%)	22	40.0	44.1	1
LD50(ppm)	21	434.8	241.4	-1
MIC50(mmol/ml)	21	55.9	48.8	-1
GII50()	21	23628.6	25104.7	-1
GI90(ug ml-1)	21	6.9	7.3	-1
Log TA100()	21	3.9	1.1	-1
Zone diameter(mM)	20	15.0	3.9	1
Hydrolysis rate()	20	0.3	0.2	1
MBC(mg/ml)	20	14.0	3.4	-1
Activity(ng)	20	220.6	444.9	-1
Selectivity()	20	80.1	135.1	1
Vmax(uM s-1)	20	0.4	0.3	1
Activity(mAU)	20	529.9	1152.3	-1
Activity(nmol/ml.min)	20	80.9	118.9	-1
Kcat/Km(10'4/s/M)	20	2.5	2.3	-1
Ka(10'5/M)	20	5.0	2.2	-1
MCC(ug.mL-1)	20	1.7	1.8	-1
logIC50()	20	0.5	1.7	1
BBC(ug.mL-1)	20	38.4	73.8	-1
pID50()	20	5.9	0.6	1
TMSW(%)	20	46.9	27.6	1
LC50(ug.mL-1)	19	19.6	16.5	-1
Growth Inhibition(mm)	18	17.1	2.5	1
MBIC90(uM)	18	70.9	33.5	-1
LD99(ug.mL-1)	18	1.3	1.8	-1
-Log C()	18	1.8	2.2	1
LD50(nM)	18	11900.0	17148.4	-1
MBC>99.9(umol/L)	18	100.7	151.3	-1
MIC(ug g-1)	18	15027.2	13948.7	-1
Zone size(mM)	18	26.7	15.2	-1
MIC50(%)	18	75.2	39.2	1
Vmax(nM s-1)	18	2.8	2.7	1
Kcat/Km(mM-1 min-1)	18	171.3	465.5	1
KA(nM)	18	37900.0	25513.2	-1

ED10(gAi/ha)	18	183.3	184.0	-1
CL(uL/min)	18	0.9	0.5	-1
CL(nl/min)	18	127.5	71.9	-1
MST(days)	18	6.6	0.9	-1
mortality(%)	17	66.5	21.6	1
MNEC(nM)	17	92647.1	20008.2	-1
T>MIC(%)	17	63.5	37.7	1
Ka(nM-1)	17	0.1	0.1	1
CC25(uM)	17	2.5	3.2	-1
Kcat/Km(10'5/s/M)	17	4.0	2.6	-1
Ka(10'6/M)	17	4.2	1.8	-1
GR20(ppm)	17	174.3	243.3	-1
Activity(M-1)	16	4790165.7	6821983.5	-1
Stability(%)	16	59.4	14.0	1
SC50(nM)	16	5000.0	5820.9	-1
Kcat/Km(10'4/M/s)	16	3.6	2.0	-1
LogP()	16	3.4	1.7	1
IC99(ug.mL-1)	16	38.9	17.8	-1
ED50(mg/kg/day)	16	15.2	10.7	-1
Drug uptake(nmol/mg)	15	21.4	42.2	1
Ratio(mM-1 s-1)	15	91.0	216.5	-1
ID50(ug ml-1)	15	42.4	15.7	-1
Activity(U/mg)	15	1.7	2.5	-1
CL(mL.min-1.g-1)	15	204189.1	232325.7	-1
MIC>99(ug ml-1)	15	0.7	0.3	-1
Kinact(10'4/M/s)	15	3.3	1.7	-1
GI(square millimeter)	14	91.8	66.4	1
Log rate constant(M-1.s-1)	14	1.8	0.9	1
Activity_index(%)	14	78.6	17.5	1
Activity(nmol/min/mg)	14	1447.2	1734.4	1
CL(mL.min-1.kg-1)	14	17.8	6.5	-1
Activity(nmol/hr)	14	102658.1	132387.3	-1
Ratio(/M/s)	14	3.8	3.2	-1
Activity(10'-5OD/s)	14	22.5	10.6	-1
IZ(mm mg-1)	14	11.1	6.5	-1
Kon(s-1)	14	3.0	3.4	1
Survival rate()	14	5.6	3.6	1
T>MIC(hr)	13	14.6	11.3	1
Activity(nmol)	13	458.8	1000.1	1
Activity(mg/L)	13	38.5	34.1	-1
LD50(mg.kg-1)	13	1620.6	1453.9	-1
Ki(10'6/M)	13	6.3	6.3	-1
Swelling(%)	13	49.9	11.3	1
MBC90(uM)	13	3.6	3.2	-1
Absorbance(nM)	12	0.6	0.5	1
MIC(ug disk-1)	12	79.2	29.1	-1
MBC(mM)	12	1.8	1.5	-1
MIC100(uM)	12	544.5	1332.1	-1

Vmax(%)	12	18.4	29.8	1
EC37(nM)	12	43.7	56.5	-1
Min concentration(ug.mL-1)	12	6.9	12.7	1
BEI(kDa-1)	12	23.8	4.5	-1
Activity(mU)	12	70.5	89.6	-1
Stabilty(%)	12	27.6	27.8	1
Activity(log CFU)	12	6.9	1.4	1
Kb(10'4/M)	12	4.0	2.3	-1
AbsAC35_uM(uM)	12	304.3	190.8	-1
M50(%)	12	14.2	33.2	1
Survival rate(%)	12	70.0	26.6	1
ED50(2mg kg-1)	12	15.9	9.1	-1
efflux_CL(ml kg-1 min-1)	12	0.0	0.0	-1
influx_CL(ml kg-1 min-1)	12	0.0	0.0	-1
ABBC(log10 CFU ml-1 hr-1)	11	53.5	3.8	-1
MIC(10'-3uM/ml)	11	60.4	40.3	-1
Phosphorolysis(%)	11	6.5	19.8	1
IC10(nM)	11	0.3	0.1	-1
Kcat/Km(10'3/M/s)	11	3.4	2.1	-1
Kcat/Km(/min/M)	11	16333.0	33630.6	1
KSV(10'5/M)	11	1.0	0.4	-1
Kq(10'12/M/s)	11	1.0	0.4	-1
K(/M/s)	11	473.4	1132.8	-1
fCmax(ug.mL-1)	11	23.7	48.6	-1
fAUC(ng.hr.mL-1)	11	92320.0	156079.0	-1
%T>MIC(%)	11	48.0	34.1	1
ED80(mg)	11	2.8	2.3	-1
Diameter(nM)	10	15.0	12.4	1
I50()	10	90.5	27.2	-1
MMC(ug.mL-1)	10	31.0	31.2	-1
Activity(min)	10	42.0	14.3	-1
Activity(s-1)	10	0.3	0.1	1
Kcat/Km(10'7/M/s)	10	2.9	1.3	-1
Ks(uM)	10	1005.9	1306.2	-1
Ki(/M/s)	10	407.9	895.1	-1
Selective index()	10	2597.9	5575.3	1
LCK()	10	2.0	0.9	-1
Rice stem length(cm)	10	2.1	0.6	1
Relative velocity(%)	9	265.4	91.4	1
Inactivation(%)	9	10.2	13.5	1
SI()	9	10.1	9.5	1
Kinact(s-1)	9	0.0	0.1	1
Delta Tm(degrees C)	9	2.1	0.7	1
Kcat/Km(microM/s)	9	1.8	2.4	-1
Vmax(umol/min/mg)	9	1.7	2.2	1
Kcat/Km(/s/M)	9	23924.1	29239.8	-1
Kb(10'7/M)	9	4.4	2.8	-1

log(1/I50)()	9	6.7	0.9	1
No. of strains()	9	2.2	1.1	-1
fCmax/MIC()	9	50.6	117.4	1
VOLSYN()	9	1558.2	883.4	-1
LD50()	9	3399.1	4428.4	-1
Ki(ng/ml)	9	1084.4	1707.8	-1
Mutation frequency()	9	20.7	21.3	-1
Activity(cm)	9	2.7	0.4	-1
I50(ppb)	8	103.5	118.1	-1
MAC(uM)	8	22.2	28.9	-1
MEC(ug.mL-1)	8	146.4	120.2	-1
LD90(uM)	8	0.2	0.2	-1
Excess zone radius(mM)	8	1.3	3.1	1
ED50(mg kg-1)	8	7.1	7.8	-1
ED100(mg kg-1)	8	13.1	13.8	-1
Dobs()	8	12.3	2.7	1
k_on(M-1.s-1)	8	283125.0	216096.4	1
permeability(um s-1)	8	0.6	0.1	1
DW()	8	14.0	3.6	1
IC0(ug.mL-1)	8	3.5	7.7	-1
IC100(ug.mL-1)	8	38.8	57.6	-1
K5(min-1)	8	0.6	0.5	-1
K6(min-1)	8	0.0	0.0	-1
Kapp(M-1)	8	16266.3	8436.3	-1
Kinact(mM)	8	20.3	32.4	1
Activity(ns)	8	3.3	2.1	-1
EC150(uM)	8	25.9	18.9	-1
RBA()	8	3.3	2.6	-1
K0.5(uM)	8	0.9	0.2	-1
Kcat/Km(/s/microM)	8	0.2	0.2	-1
K(/s)	8	1.7	2.0	1
Kcat/Km(/min/microM)	8	0.9	0.5	-1
Fu()	8	0.6	0.3	1
Safety index()	8	30.4	24.5	1
log10CFU/ml/day()	8	0.3	0.3	1
GR10(gAi/ha)	8	50.0	24.2	-1
MBC(umol)	8	39.1	23.9	-1
NSR survival rate()	8	7.6	2.6	1
Kinact(10'5/M/s)	8	2.2	0.8	-1
IC12(ug)	8	20.5	17.0	-1
Drug metabolism(nmol/min)	8	3.9	4.3	-1
Revertant colonies per plate()	8	707.8	381.7	1
CFU(%)	8	55.5	27.2	-1
Activity(pg ml-1)	8	2059.5	1734.3	-1
MIC(umol/uL)	7	0.2	0.1	-1
Activity(pmol)	7	10.2	10.0	1
Growth Inhibition(%)	7	79.0	33.8	1

PD50(ug.mL-1)	7	5.5	4.7	-1
k cat/Km(M-1.s-1)	7	6200.5	9459.0	1
K1(nM)	7	7664.8	17313.4	-1
IC100(nM)	7	21428.6	17978.2	-1
Km(uM-1)	7	73.9	151.4	1
Kcat(app)(/s)	7	17.9	18.5	-1
Drug metabolism(uM)	7	11.5	6.0	-1
Vmax(mM/min)	7	0.2	0.1	1
Vm(uM min-1 (mg of protein)-1)	7	5.2	3.7	1
MST(day)	7	30.8	10.0	-1
MBC(ug)	7	164.3	54.3	-1
Activity(/nmol)	7	14.8	32.5	-1
Kcat(hr-1)	6	0.4	0.4	1
ED50(ng ml-1)	6	49.8	20.0	-1
Activity(nmol/mg/min)	6	67.7	51.5	1
MBIC(ug.mL-1)	6	38.3	29.3	-1
MIC+/MIC-()	6	4.1	3.6	-1
ABBC(log10CFU/ml.hr)	6	1.4	2.5	1
AUBC(log10CFU/ml.hr)	6	214.5	2.4	1
LD90(ug ml-1)	6	4.7	1.6	-1
LD50(ug ml-1)	6	1.7	0.7	-1
Activity(mm mg-1 ml-1)	6	0.0	0.0	1
MIC95()	6	166.7	69.0	-1
RVmax()	6	30.9	37.8	1
GI(mM)	6	20.0	3.1	-1
Activity index()	6	0.8	0.9	1
Kkmax(hr-1)	6	4.4	1.2	1
Ks(hr-1)	6	2.1	0.4	1
Cell growth()	6	0.4	0.5	1
C50(M)	6	3485.1	4268.3	-1
Rate of hydride transfer(s-1)	6	17.9	14.8	1
K2(nM)	6	5116.5	10303.6	-1
Kic(nM)	6	7906.3	15774.6	1
Relative activation(%)	6	107.5	30.1	1
Vmax(pmol min-1)	6	594.3	475.4	1
Vmax(nmol/min)	6	8.3	9.4	1
Ka(uM-1)	6	122.5	119.6	1
Activity(umol)	6	30.2	46.6	-1
Vmax(nmol/mg/min)	6	120.8	93.3	1
IC100(umol/L)	6	31.7	13.8	-1
deltaT(degrees C)	6	2.0	0.0	1
Kcat/Km(/uM/s)	6	7.4	5.6	-1
K(nM)	6	203.1	318.2	-1
MTD50(ug.mL-1)	6	24.0	18.1	-1
ED99(mg kg-1)	6	75.0	119.2	-1
Emax(mg kg-1)	6	292.5	311.3	-1
MST()	6	27.6	7.7	1

Vmax/Km(/min)	6	0.3	0.4	1
Activity(ug/g)	6	74.5	54.1	-1
fT>MIC(hr)	6	1.8	0.5	-1
Cell aggregates()	6	57.2	22.5	1
Slope(mutants ug-1)	6	1.5	1.3	-1
LD90(ppm)	5	60.0	11.2	-1
EC95(ug.mL-1)	5	23.9	34.9	-1
MBEC(ug.mL-1)	5	60.2	89.7	-1
Cmin(ug.mL-1)	5	4.1	1.8	-1
Activity(U)	5	233.2	199.1	1
ID50(mg ml-1)	5	2.1	1.0	-1
Activity(mg/ml)	5	0.8	0.6	-1
MBC90(ug ml-1)	5	425.4	718.9	-1
K+2(min-1)	5	0.3	0.2	-1
Substrate efficiency(%)	5	50.6	32.9	1
kinact(min-1)	5	0.0	0.0	1
Vmax(mM min-1)	5	2.0	1.9	1
K(mM)	5	1.8	2.8	-1
Kcat/Km(M-1 hr-1)	5	2440.6	1833.7	1
Vmax(mmol min-1 mg-1)	5	2.8	2.5	1
Vmax(microM/min/mg)	5	485.6	233.3	1
Vmax(microM/s)	5	19.9	35.2	1
ID50(uM)	5	279.8	341.6	-1
Activity(RLU)	5	125.4	109.7	-1
Kinact(/min)	5	0.1	0.1	1
Kcat/Km(nM-1)	5	0.0	0.0	1
Vm(uM min-1 mg-1)	5	7.2	2.5	1
EC90/MIC()	5	2.2	2.7	1
AUC(uM.hr)	5	89.7	82.5	-1
GR80(gAi/ha)	5	56.2	45.1	-1
MBEC50(uM)	5	27.2	12.6	-1
AAC(delta log10 CFU (ml hr)-1)	5	-5.5	11.9	1
Challenge dose()	5	379360.0	280224.0	-1
IC50(%)	5	47.4	33.2	1
MIC95(ug ml-1)	4	20.0	15.5	-1
AG80(%)	4	77.3	19.1	1
Ratio ED50()	4	3.6	1.4	-1
AD50(ug.mL-1)	4	46.4	23.1	-1
GI90(M)	4	0.0	0.0	-1
MBC(umol/L)	4	6.3	6.3	-1
MIC100(umol/ml)	4	128.7	138.3	-1
RFU()	4	156.3	16.5	-1
MBC50(ug ml-1)	4	390.6	572.9	-1
Ratio(nM)	4	0.9	0.4	1
K inact(min-1)	4	0.8	0.6	1
Efficiency(%)	4	65.0	23.8	1
Vmax(c.p.m.)	4	1318.5	515.3	1

Ki app(mole fraction)	4	0.1	0.2	-1
K(M-1)	4	2961250.0	2644719.5	1
Drug uptake(mg ml-1)	4	1.8	1.2	-1
Vmax/Km(%)	4	9.0	5.7	1
Kcat/Km(10'2/M/s)	4	5.4	2.1	-1
pIC50()	4	4.1	0.0	1
Ka(/uM)	4	9.1	11.2	-1
Kcat/Km(10'-3/microM/s)	4	2.9	1.2	-1
Kb(10'8/M)	4	2.3	1.4	-1
K(10'6/M)	4	14.1	9.8	-1
ICmax(%)	4	51.3	26.9	1
RU()	4	15.5	8.5	-1
Kdiss(/s)	4	0.0	0.0	1
Ka(10'4/M/s)	4	3.9	2.8	-1
VmaxH(pmol/min)	4	137.3	88.5	1
KmH(uM)	4	1444.9	1829.2	-1
Kinact(hr-1)	4	169.8	229.3	1
EC90()	4	0.3	0.2	-1
EC99()	4	62.3	37.0	-1
AUC(ug.mL-1)	4	77.2	56.9	-1
Log10 cfu(mg)	4	2.1	0.6	1
Post antibiotic effect(days)	4	12.4	4.5	1
Vd(L.kg-1)	4	0.8	0.5	1
Kel(hr-1)	4	1.3	0.7	1
INH(ug ml-1)	4	8.8	1.9	-1
Activity(hr-1)	4	2.0	2.3	1
Delta log10CFU(ml-1)	4	-1.1	0.2	1
Challenge dose(cells mouse-1)	4	1000000.0	0.0	-1
Vd/F(L.kg-1)	4	5.7	2.3	1
CL/F(mL.min-1.kg-1)	4	65.4	54.4	-1
Kcat/Km(ug.mL-1)	4	0.1	0.0	1
Activity(c.p.m. min-1)	4	1375.0	1006.2	-1
Log10 cfu(ml-1)	4	5.0	1.2	1
Km(ug.mL-1)	4	42.3	5.7	-1
Vmax(ug min-1 kg-1)	4	0.8	0.0	1
Vmax(pmol mg-1 min-1)	3	1073.3	258.9	1
I50(ppm)	3	1.4	0.9	-1
LD50(ug.mL-1)	3	6.3	2.3	-1
Time(day)	3	19.6	1.1	1
ILS(%)	3	18.7	0.8	1
Activity(nM-1)	3	17.8	11.3	-1
Dioxygen uptake rate(uM min-1)	3	20.0	14.0	1
T1/2 regen(s)	3	2672.3	1547.2	1
Difference in retention time(min)	3	1.3	1.3	1
Concentration(nM)	3	36.7	16.3	-1

Drug uptake(pmol)	3	173.3	63.8	-1
I50 M()	3	1046.7	658.2	-1
Ki(TS)	3	5.5	6.7	-1
Vmax(uM min-1)	3	1.6	0.7	1
Kapp(nM)	3	2390.0	1429.6	1
Ratio pIC50()	3	33700.0	40601.5	1
F(%)	3	60.2	12.1	1
Kiuc(nM)	3	129.3	36.6	-1
Ratio(uM-1 s-1)	3	9.0	4.3	1
Kcat/Km(10'-4/uM/s)	3	5.0	1.6	-1
KSV(10'4/M)	3	1.4	0.1	-1
Kq(10'12L/mol/s)	3	1.4	0.1	-1
Ka(10'6/M/s)	3	2.7	2.0	-1
Retention_time(min)	3	12.4	6.4	1
Ratio(/uM/min)	3	0.1	0.1	-1
ft1/2(hr)	3	1.3	0.2	-1
PB(%)	3	43.7	27.3	1
IC50()	3	27.5	21.4	-1
ID50(mg.kg-1)	3	3.6	0.8	-1
Drug uptake(ng)	3	102.0	18.7	-1
ED30(mg kg-1)	3	32.7	36.4	-1
Activity(10'7CFU)	3	1.8	0.3	-1
MIC90(%v/v)	3	0.0	0.0	-1
Drug uptake(ppm)	3	8.0	7.3	-1
Activity(10'-7 ug/ml)	3	3.6	2.2	-1
Activity(10'-6 ug/ml)	3	2.1	0.2	-1
Activity(mg.min/L)	3	350.0	398.1	-1
Kinact(10'3/M/s)	3	5.5	2.4	-1
IC12(nM)	3	315800.0	49566.7	-1
IC80(ug.mL-1)	3	13.5	11.6	-1
MIC100(nM)	3	25540.0	29676.4	-1
K inact/Ki(uM-1 min-1)	3	10.8	11.1	1
MBEC(nM)	2	4000.0	0.0	-1
MBIC(nM)	2	220.0	0.0	-1
Range(ug.mL-1)	2	6.0	1.6	1
Growth response(%)	2	0.0	0.0	1
RHR()	2	53.0	43.3	1
ILB()	2	1.2	0.0	-1
Zone reduction(%)	2	19.5	6.1	1
pKa2()	2	7.0	0.8	1
pKa1()	2	4.0	0.8	1
Activity(mg/kg/day)	2	4.0	0.8	1
ID50(10'-7M)	2	2.5	1.2	-1
Log(log (1/M MIC))	2	6.5	0.1	1
GI(uM)	2	52.0	39.2	-1
K inact/Ki(M-1.s-1)	2	821.0	314.4	1
k cat(min-1)	2	970.0	498.1	1
Vmax(umol min-1 mg-1)	2	9.4	1.3	1

Fluorescence Intensity()	2	204651.0	18193.2	1
Fluorescence Intensity(%)	2	3.9	1.2	1
KR(nM)	2	496500.0	73076.4	-1
Km(s-1)	2	0.4	0.2	-1
Ratio(min-1)	2	7.5	6.1	-1
Activity(hr)	2	10.0	6.5	-1
Ki/Km()	2	0.1	0.0	-1
K(10'5/M)	2	2.9	0.4	-1
CL_renal(mL.min-1.kg-1)	2	3.7	0.4	-1
Kcat/Km(10'2 uM/s)	2	1.0	0.0	-1
Kcat/Km(10'8/M/s)	2	1.7	0.0	-1
Kcat/Km(s-1)	2	0.0	0.0	-1
Cmin(mg/L)	2	0.9	0.2	-1
log Ks()	2	4.6	0.5	1
Ka(10'7/M)	2	1.3	0.0	-1
RUmax(%)	2	29.5	3.7	1
Kcat/Km(/M/s)	2	12580.0	7201.5	-1
Ka(10'4/M)	2	3.2	0.4	-1
Activity(10'-8M)	2	32500.3	26535.9	-1
Vmax(pmol/min/ug)	2	13.5	2.0	1
Activity(g)	2	1.0	0.0	-1
Kcat(10'5/s)	2	7.1	0.0	-1
Activity(umol min-1 mg-1)	2	7.5	0.1	-1
Tmax(ug hr ml-1)	2	104.6	75.4	-1
AUC(hr)	2	0.8	0.2	-1
Hydrolysis(%)	2	29.0	4.9	1
Log CFU()	2	6.2	0.1	1
Kgmax(log10CFU ml-1 hr-1)	2	0.0	0.0	1
C50g(ug.mL-1)	2	967.0	787.9	-1
Hg()	2	2.5	1.7	-1
Kkmax(log10CFU ml-1 hr-1)	2	0.0	0.0	1
C50k(ug.mL-1)	2	15.0	6.0	-1
Hk()	2	23.4	17.9	1
Kb(10'5/M)	2	1.4	0.1	-1
Ka(hr-1)	2	5.6	1.8	1
MBC(mg kg-1)	2	100.0	0.0	-1
GI(ug.mL-1)	2	256.0	0.0	-1
Activity(Da)	2	410.3	27.4	-1
INH(ug.mL-1)	2	0.7	0.4	-1
CFU(10'8/ml)	2	0.9	0.1	-1
ID50(uM/ml)	2	0.4	0.1	-1
MBIC(ug ml-1)	2	11.8	5.5	-1
Activity(10'-5 ug/ml)	2	1.0	0.0	-1
MTD(mg Kg-1)	2	230.0	138.8	-1
Max fold increase()	2	37.0	1.6	1
MIC(ug hr ml-1)	2	64.0	0.0	-1

Activity(log10 CFU ml-1)	2	0.0	0.0	-1
Cmax(mg Kg-1)	2	3.0	0.0	-1
ED50(ml/kg)	2	23.3	0.0	-1
Activity(mg kg-1)	2	80.0	0.0	-1
Kact(min-1)	2	0.2	0.0	-1
IC50(ppm)	1	10.0	0.0	-1
LD50(mM)	1	0.1	0.0	-1
LD100(ppm)	1	0.1	0.0	-1
Activity(days)	1	10.0	0.0	1
TC50(nM)	1	16000.0	0.0	-1
TC50(uM)	1	2000.0	0.0	-1
Range()	1	0.3	0.0	1
MED(uM)	1	0.7	0.0	-1
CFU(10'6/ml)	1	5.7	0.0	-1
PD50(mg kg-1 p.o.)	1	0.8	0.0	-1
pMIC(ug.mL-1)	1	0.1	0.0	1
ID50(10'-4M)	1	3.0	0.0	-1
CFU(10'3/ml)	1	2.7	0.0	-1
CFU(10'2/ml)	1	3.7	0.0	-1
AD50(%)	1	70.0	0.0	1
Imax(ug.mL-1)	1	2.0	0.0	-1
ICmax(ug.mL-1)	1	2048.0	0.0	-1
Potency(%)	1	1.0	0.0	1
MBIC90(ug ml-1)	1	2.2	0.0	-1
MIC(%)	1	4.0	0.0	1
K1(mM)	1	1.7	0.0	-1
K2(mM)	1	1.9	0.0	-1
K inact(s-1)	1	0.0	0.0	1
K inact(M-1 min-1)	1	0.1	0.0	1
K+2/Ki(uM min-1)	1	0.1	0.0	-1
Kcat(mM)	1	0.3	0.0	1
LD100(ng)	1	100.0	0.0	-1
K(nM-1)	1	0.0	0.0	1
Vmax(uM)	1	19.0	0.0	1
XL50(nM)	1	0.9	0.0	-1
Vdss(L.kg-1)	1	3.3	0.0	-1
Ratio LD50/ED50()	1	200.0	0.0	-1
Kiuc(10'3nM)	1	1.1	0.0	-1
Kdiss(min-1)	1	0.0	0.0	1
Ks(mM)	1	1.1	0.0	-1
Kcat/Km(10'-2/microM/s)	1	3.9	0.0	-1
Bmax(pg/mm2)	1	67.0	0.0	-1
Bmax(pmol)	1	45.8	0.0	-1
Kcat(10'6/s)	1	4.1	0.0	-1
Kcat(10'7/s)	1	1.4	0.0	-1
Ka(10'-7M)	1	1.3	0.0	-1
Kb(10'9/M)	1	2.3	0.0	-1
Kb(10'6/M)	1	5.3	0.0	-1

K(10'9/M)	1	2.5	0.0	-1
K(10'11/M)	1	3.2	0.0	-1
Ka(10'5/M/s)	1	10.0	0.0	-1
K(10'7/M)	1	2.3	0.0	-1
K(10'8/M)	1	9.7	0.0	-1
alphaKi(mM)	1	28.0	0.0	-1
Kmax(/hr)	1	124.6	0.0	1
INH(nM)	1	5.0	0.0	-1
alphaKi(uM)	1	3.0	0.0	-1
MIC90(%)	1	1.0	0.0	-1
Vmax(pM min-1 mg-1)	1	4.3	0.0	1
Range of MIC(ug.mL-1)	1	1.0	0.0	-1
Vmax(U mg-1)	1	0.8	0.0	-1
MIC(mg)	1	1.0	0.0	-1
Cmax(nM)	1	700.0	0.0	-1
CL(L hr-1)	1	99.0	0.0	-1
Vc(L)	1	430.9	0.0	-1
Activity(mg L-1)	1	50.0	0.0	-1
EC90(mg ml-1)	1	0.0	0.0	-1
EC99(mg ml-1)	1	0.4	0.0	-1
EC99(uM)	1	18.3	0.0	-1
Activity(10'7/g)	1	9.0	0.0	-1
Activity(10'12L/mol/s)	1	1.6	0.0	1
Activity(10'10L/mol/s)	1	2.0	0.0	1
MIC97(ug.mL-1)	1	0.0	0.0	-1
WCC90(ug ml-1)	1	1.0	0.0	-1
Drug metabolism(nM)	1	0.5	0.0	-1
MBC(ug cm-3)	1	2.0	0.0	-1
CFU(ml)	1	0.0	0.0	-1
Activity(10'-8 ug/ml)	1	9.6	0.0	-1
Activity(10'8CFU/ml)	1	9.8	0.0	-1
C50k(mg/L)	1	0.7	0.0	-1
f%T>MIC(%)	1	54.0	0.0	1
Vmax/Km()	1	7900.0	0.0	1
Ratio(nM-1)	1	0.0	0.0	1
Vc/F(l)	1	0.0	0.0	1
CL/F(ml/min)	1	1.3	0.0	-1
Vd(l)	1	0.0	0.0	1
Kiuc(mM)	1	2.0	0.0	-1
Kinact(10'2/M/s)	1	8.1	0.0	-1
IC40(uM)	1	17.0	0.0	-1
IC20(uM)	1	7.5	0.0	-1
Ki inact(min-1)	1	1.5	0.0	1
MFC(nM)	1	150000.0	0.0	-1
MFC(mg ml-1)	1	0.1	0.0	-1
Kcat(nM)	1	2550.0	0.0	-1
Vmax/Km(M-1.s-1)	1	7100.0	0.0	1
Vmax(s-1)	1	1.3	0.0	1

Survival(day)	1	8.1	0.0	1

Table S02. Details of the metabolic networks of >40 organisms

Organism Name	Symbol	N[a]	L_{in} [b]	L_{out} [b]	Organism Name	Symbol	N[a]	L_{in} [b]	L_{out} [b]
Aeropyrum pernix	AP	204	588	575	*Chlorobium tepidum*	CL	389	1097	1062
Archaeoglobus fulgidus	AG	496	1527	1484	*Rhodobacter capsulatus*	RC	670	2174	2122
Methanobacterium thermoautotrophicum	TH	430	1374	1331	*Rickettsia prowazekii*	RP	214	510	504
Methanococcus jannaschii	MJ	424	1317	1272	*Neisseria gonorrhoeae*	NG	406	1298	1270
Pyrococcus furiosus	PF	316	901	867	*Neisseria meningitidis*	NM	381	1212	1181
Pyrococcus horikoshii	PH	323	914	882	*Campylobacter jejuni*	CJ	380	1142	1115
Aquifex aeolicus	AA	419	1278	1249	*Helicobacter pylori*	HP	375	1181	1144
Chlamydia pneumoniae	CQ	194	401	391	*Escherichia coli*	EC	778	2904	2859
Chlamydia trachomatis	CT	215	479	462	*Salmonella typhi*	TY	819	3008	2951
Synechocystis sp.	CY	546	1782	1156	*Actinobacillus actinomycetemcomitans*	AB	395	1202	1166
Porphyromonas gingivalis	PG	424	1192	1221	*Haemophilus influenzae*	HI	526	1773	1746
Mycobacterium bovis	MB	429	1247	1244	*Pseudomonas aeruginosa*	PA	734	2453	2398
Mycobacterium leprae	ML	422	1271	1823	*Treponema pallidum*	TP	207	562	555
Mycobacterium tuberculosis	MT	587	1862	2741	*Borrelia burgdorferi*	BB	187	442	438
Bacillus subtilis	BS	785	2794	1218	*Thermotoga maritima*	TM	338	1004	976
Enterococcus faecalis	EF	386	1244	1578	*Deinococcus radiodurans*	DR	815	2870	2811
Clostridium acetobutylicum	CA	494	1624	525	*Emericella nidulans*	EN	383	1095	1081
Mycoplasma genitalium	MG	209	535	466	*Saccharomyces cerevisiae*	SC	561	1934	1889
Mycoplasma pneumoniae	MP	178	470	1298	*Caenorhabditis elegans*	CE	462	1446	1418
Streptococcus pneumoniae	PN	416	1331	1277	*Oryza sativa*	OS	292	763	751
Streptococcus pyogenes	ST	403	1300		*Arabidopsis thaliana*	AT	302	804	789

[a] N = number of nodes (metabolites), [b] L = input-output links (metabolic reactions), according to Jeong *et al.* [10]

Table S03. Average values of f_k for the metabolic networks of >40 organisms

Org.[a]	Node degrees [b]			Markov Linear Indices [c]					
	δ	δ_{in}	δ_{out}	f_0	f_1	f_2	f_3	f_4	f_5
AA	0.10	0.10	0.10	0.10	-0.65	-0.59	-0.60	-0.52	-0.50
AB	0.04	0.04	0.04	0.04	0.20	0.08	0.09	0.03	-0.01
AG	-0.05	-0.05	-0.05	-0.05	-0.66	-0.68	-0.69	-0.67	-0.65
AP	-0.06	-0.06	-0.06	-0.06	0.15	0.04	0.12	0.03	0.03
AT	-0.79	-0.79	-0.79	-0.79	-0.64	-0.72	-0.74	-0.80	-0.82
BB	-2.13	-2.13	-2.13	-2.13	-0.51	-0.64	-0.65	-0.71	-0.71
BS	1.09	1.09	1.09	1.09	3.23	3.03	2.83	2.70	2.55
CA	0.69	0.69	0.69	0.69	-0.60	-0.42	-0.42	-0.27	-0.24
CE	0.58	0.58	0.58	0.58	-0.53	-0.46	-0.51	-0.45	-0.43
CJ	0.06	0.06	0.06	0.06	-0.47	-0.35	-0.40	-0.32	-0.31
CL	-0.26	-0.26	-0.26	-0.26	0.85	0.95	1.13	1.14	1.12
CQ	-3.11	-3.11	-3.11	-3.11	0.25	0.25	0.17	0.06	-0.01
CT	-2.56	-2.56	-2.56	-2.56	-0.69	-0.98	-1.00	-1.17	-1.18
CY	0.81	0.81	0.81	0.81	1.06	1.08	1.23	1.15	1.18
DR	1.04	1.04	1.04	1.04	0.44	0.29	0.47	0.37	0.39
EC	1.43	1.43	1.43	1.43	0.57	0.78	0.78	0.94	0.94
EF	0.67	0.67	0.67	0.67	-0.64	-0.49	-0.47	-0.32	-0.27
EN	-0.04	-0.04	-0.04	-0.04	-0.51	-0.59	-0.63	-0.70	-0.72
HI	0.85	0.85	0.85	0.85	1.29	0.96	0.98	0.81	0.78
HP	0.65	0.65	0.65	0.65	0.24	0.50	0.60	0.82	0.84
MB	-0.13	-0.13	-0.13	-0.13	-0.63	-0.80	-0.82	-0.90	-0.91
MG	-1.29	-1.29	-1.29	-1.29	-0.66	-0.90	-0.93	-1.08	-1.10
MJ	0.12	0.12	0.12	0.12	-0.65	-0.62	-0.63	-0.58	-0.57
ML	0.04	0.04	0.04	0.04	-0.56	-0.70	-0.75	-0.83	-0.85
MP	-0.96	-0.96	-0.96	-0.96	-0.59	-0.86	-0.92	-1.10	-1.14
MT	0.41	0.41	0.41	0.41	1.22	1.24	1.38	1.39	1.43
NG	0.51	0.51	0.51	0.51	-0.58	-0.46	-0.49	-0.41	-0.41
NM	0.55	0.55	0.55	0.55	-0.56	-0.24	-0.22	0.04	0.10
OS	-0.96	-0.96	-0.96	-0.96	-0.64	-0.69	-0.69	-0.69	-0.68

PA	0.76	0.76	0.76	0.76	3.51	3.42	2.97	2.78	2.73
PF	-0.24	-0.24	-0.24	-0.24	-0.67	-0.88	-0.90	-1.02	-1.05
PG	-0.51	-0.51	-0.51	-0.51	-0.66	-0.47	-0.44	-0.25	-0.20
PH	-0.36	-0.36	-0.36	-0.36	-0.60	-0.70	-0.75	-0.80	-0.81
PN	0.57	0.57	0.57	0.57	-0.63	-0.41	-0.39	-0.20	-0.15
RC	0.54	0.54	0.54	0.54	1.97	1.93	2.43	2.37	2.51
RP	-1.46	-1.46	-1.46	-1.46	-0.60	-0.86	-0.89	-1.05	-1.07
SC	1.46	1.46	1.46	1.46	0.16	0.38	0.40	0.51	0.57
ST	0.67	0.67	0.67	0.67	-0.34	-0.38	-0.38	-0.38	-0.37
TH	0.49	0.49	0.49	0.49	-0.62	-0.46	-0.47	-0.36	-0.35
TM	0.05	0.05	0.05	0.05	-0.51	-0.47	-0.49	-0.46	-0.47
TP	-0.72	-0.72	-0.72	-0.72	-0.27	-0.42	-0.47	-0.58	-0.59
TY	1.34	1.34	1.34	1.34	0.52	0.56	0.39	0.31	0.28
YP	0.07	0.07	0.07	0.07	-0.00	0.76	0.75	1.16	1.14

[a] Org = organism, Organisms names are in two letters code and metabolite names in numeric code, according to Jeong *et al.* [10].

[b] Average node degrees, δ. [c] All indices have been standardized to z-scaled values to avoid scale errors in visual comparison.

Table S04. Conditions includes in ChEMBL Dataset of Antibacterial Drugs *vs* MRN analysis.

Cond Number	Name	Description	Items	Main categories
c_0	Biological Activity	Parameter determined for biological activity vij (MIC, MBC, etc.) per se	802	MIC ($\mu g.mL^{-1}$, IC50(nM), IZ(mm), *etc*
c_1	Protein Accession	Identifier for a specific protein in a database. (1039 items)	1039	Various
c_2	Assay Organism	Assay Organism in experiment	335	Various
c_3	Assay Strain	Specific Strain of assay organism	4670	Various
c_4	MN Microorganism	MN Microorganism specie	37	More detail (Table **S01**)
c_5	Target Type	Different target types	12	Organism, Single Protein, Unchecked, Cell-Line, Nucleic-Acid, Protein Complex, Admet, Protein Family, No Target, Tissue, Protein Complex Group, Protein-Protein Interaction
c_7	Target Mapping	Mappings to ChEMBL targets	9	Non-molecular, Protein Unassigned, Homologous protein, Multiple proteins, Multiple homologous proteins, Homologous protein complex, Molecular (non-protein), Protein complex
c_8	Confidence Score	This value reflects both the type of target assigned to a particular assay and the confidence that the target assigned is the correct target for that assay	9	0, 1, 3 to 9*
c_9	Curated	Level of Data curated in ChEMBL database	3	Intermediate, Autocuration, Expert
c_{10}	Assay Type	Type of data measuring or assay in ChEMBL database	5	Binding (B), Functional (F), ADMET (A), Physicochemical (P), Unclassified (U)

* Value definition: 0 Default value (Target assignment has yet to be curated), 1 Target assigned is non-molecular , 3 Target assigned is molecular non-protein target, 4 Multiple homologous protein targets may be assigned, 5 Multiple direct protein targets may be assigned, 6 Homologous protein complex subunits assigned , 7 Direct protein complex subunits assigned, 8 Homologous single protein targets assigned , 9 Direct single protein targets assigned

Table S05. Linear index based on atoms descriptors included in the model

Code	Descriptor Code TOMOCOMD-CARDD	Description
d_{14}	N1_F_AB_nCi_2_NS2_C_KA_e_MAS	N1 -> Manhattan Distance Invariant/F -> Linear Indices/AB > Atom based Level/nCi -> Non-chiral indices/2 -> Duplex/NS -> Non Stochastic Matrix Order: 2/C -> C atoms in aliphatic chain/KA -> Means keep all elements in the matrix form (entire matrix)/e -> Electronegativity/MAS -> QuBiLs-MAS
d_{15}	N1_F_AB_nCi_2_NS3_C_KA_e_MAS	N1 -> Manhattan Distance Invariant/F -> Linear Indices/AB -> Atom based Level/nCi -> Non-chiral indices/2 -> Duplex/NS -> Non Stochastic Matrix Order: 3/C -> C atoms in aliphatic chain/KA -> Means keep all elements in the matrix form (entire matrix)/e -> Electronegativity/MAS -> QuBiLs-MAS
d_{00}	N1_F_AB_nCi_2_NS0_T_KA_e_MAS	N1 -> Manhattan Distance Invariant/F -> Linear Indices/AB -> Atom based Level2 -> Duplex/NS -> Non Stochastic Matrix Order: 0/T -> Total (Global) indices/KA -> Means keep all elements in the matrix form (entire matrix)/e -> Electronegativity/MAS -> QuBiLs-MAS
d_{01}	N1_F_AB_nCi_2_NS1_T_KA_e_MAS	N1 -> Manhattan Distance Invariant/F -> Linear Indices/AB -> Atom based Level/nCi -> Non-chiral indices/2 -> Duplex/NS -> Non Stochastic Matrix Order: 1/T -> Total (Global) indices/KA -> Means keep all elements in the matrix form (entire matrix)/e -> Electronegativity/MAS -> QuBiLs-MAS
d_{02}	N1_F_AB_nCi_2_NS2_T_KA_e_MAS	N1 -> Manhattan Distance Invariant/F -> Linear Indices/AB -> Atom based Level/nCi -> Non-chiral indices/2 -> Duplex/NS -> Non Stochastic Matrix Order: 2/T -> Total (Global) indices/KA -> Means keep all elements in the matrix form (entire matrix)/e -> Electronegativity/MAS -> QuBiLs-MAS
d_{03}	N1_F_AB_nCi_2_NS3_T_KA_e_MAS	N1 -> Manhattan Distance Invariant/F -> Linear Indices/AB -> Atom based Level/nCi -> Non-chiral indices/2 -> Duplex/NS -> Non Stochastic Matrix Order: 3/T -> Total (Global) indices/KA -> Means keep all elements in the matrix form (entire matrix)/e -> Electronegativity/MAS -> QuBiLs-MAS
